# De novo genome assembly, inversion detection, and worldwide adaptation on the invasive species *Styela plicata*

**DOI:** 10.1038/s41598-025-24574-8

**Published:** 2025-11-18

**Authors:** Carles Galià-Camps, Tilman Schell, Cinta Pegueroles, Damian Baranski, Alexander Ben Hamadou, Mathilde Horaud, Adrià Antich, Xavier Turon, Marta Pascual, Carola Greve, Carlos Carreras

**Affiliations:** 1 Departament de Genètica, Microbiologia i Estadística, Universitat de Barcelona, Avinguda Diagonal 643, 08028 Barcelona, Spain; 2 Institut de Recerca de la Biodiversitat (IRBio), Universitat de Barcelona (UB), Barcelona, Spain; 3 LOEWE Centre for Translational Biodiversity Genomics (LOEWE-TBG), Senckenberganlage 25, 60325 Frankfurt am Main, Germany; 4 Senckenberg Forschungsinstitut und Naturmuseum, Senckenberganlage 25, 60325 Frankfurt am Main, Germany; 5 Department of Genetics and Microbiology, Universitat Autònoma de Barcelona, 08193 Bellaterra, Barcelona, Spain; 6 Institute of Biotechnology and Biomedicine, Universitat Autònoma de Barcelona, 08193 Bellaterra, Barcelona, Spain; 7 Faculty of Biosciences, Fisheries and Economics, The Norwegian College of Fishery Science, UiT The Arctic University of Norway, Tromsø, Norway; 8 Centre for Advanced Studies of Blanes (CEAB), CSIC, Accés Cala St. Francesc 14, 17300 Blanes, Girona Spain

**Keywords:** Computational biology and bioinformatics, Ecology, Ecology, Evolution, Genetics, Ocean sciences

## Abstract

Biological invasions are a major threat to biodiversity. However, genomic resources of invasive species are scarce, compromising the study of their invasive success and our ability to mitigate their effects. Here, we assemble and annotate the reference chromosome-level genome of the invasive ascidian *Styela plicata*, and complement it with whole genome sequencing data from 24 individuals worldwide. We developed and validated the novel method “individual Detection of linkage by Genotyping” (iDlG), that allowed identifying four large chromosomal inversions and assigning karyotypes at the individual level. The four inversions are polymorphic throughout the species’ distribution range and are enriched with genes that potentially influence fitness in estuarine and harbor environments, where *Styela plicata* thrives. It was only after we removed the inversions that we could detect clear population structuring, both between and within oceans, driven by several candidate adaptive genes involved in osmoregulation and other functions. Moreover, we recovered three major mitogenomic lineages, two of them globally sympatric and one specific to a single population. Interestingly, mitochondrial lineages show associations with nuclear genes likely involved in correct mitochondrion distribution during cell division. Our study highlights the importance of generating annotated reference genomes and combining them with whole genome sequencing data across whole distribution ranges to identify species’ structural and sequence variation for understanding complex evolutionary processes.

**Supplementary Information:**

The online version contains supplementary material available at 10.1038/s41598-025-24574-8.

## Introduction

We are facing a global biodiversity crisis due to climate change^1,2^. As a result, species worldwide adapt to the changing environments, migrate to suitable areas^3^, or become extinct^4^. These contemporary processes provide unique opportunities to observe evolution in action over relatively short periods of time in a plethora of different organisms^5^. Consequently, the study of current biodiversity shifts is of the utmost interest, as it opens up the possibility of assessing adaptation to rapidly changing environments, with genomes being the printed legacy of the evolutionary signals that witness the action of natural selection^6^. Recent advances in genomics have boosted research for wildlife management in non-model organisms^7^, and initiatives have emerged to generate reference genomes for all species worldwide^8,9^. Nevertheless, a single reference genome is insufficient to capture the species-specific diversity in terms of structural and sequence variants^10^.

The inclusion of multiple genomes of the same species along its distribution range to obtain the species’ genome diversity is needed to fully understand evolutionary forces in action^11,12^. To combine and compare genomic data from multiple specimens allow a complete evaluation of polymorphisms to address, with improved accuracy, evolutionary questions that are critical for wildlife management efforts^13^. These include single nucleotide polymorphisms (SNPs), which are widely used for genomic studies, but also structural variants that are essential for understanding species’ evolutionary processes such as linkage regions, which are block regions in the genome that do not recombine and co-evolve. Among linkage regions, chromosomal inversions have been traditionally studied, and have been demonstrated to play key roles in adaptive evolution, speciation, or the generation of sexual chromosomes^14^. Consequently, the identification of chromosome inversions has become an important focus of current genomic research, as also developing methods to identify them based on cost-effective whole genome sequencing (WGS) Illumina reads. Inversions (and other linked regions) are often detected by comparing the genomic data from pre-defined groups with discrete morphotypes or ecotypes through sliding-windows based on F_ST_ analyses, which reveal regions with extremely high values of differentiation^15^. However, the presence of inversions is not always reflected by discrete and morphologically recognizable phenotypes, and thus the F_ST_ approximation cannot be used since it requires the prior identification of the groups to carry out the analyses. Linkage Disequilibrium analyses, based on the correlation of genotypes across chromosomes, can identify the presence of inversions across multiple samples without phenotype differentiation^15^. High correlation values indicate linked regions, while low values indicate regions that freely recombine. Even so, this approach does not allow individual karyotype assignment. A common strategy to solve the assignment limitation is to carry out local Principal Component Analyses (PCA) or Multi-Dimensional Scaling (MDS) approaches using the markers of the region previously identified as an inversion, which will show up as three clearly delimited groups on the first axis, corresponding to individuals homokaryotpes for each of the two possible chromosomal arrangements or heterokaryotype (one chromosome with each arrangement)^15^. However, the resulting signal is only clear when only one inversion is analyzed at a time^16–18^. In summary, to date, there is no software capable of effectively handling the identification of multiple inversions and other linked regions without any prior phenotypic or ecological information, determining their area of influence, and carrying out an assignment of each inversion karyotype to each individual included into the analysis, all at once.

In the context of the ongoing global biodiversity crisis, invasive species are one of the most threatening factors worldwide, and are considered to be one of the main causes of species extinction^19,20^. When settled in a new habitat, they may outcompete native species, displacing them to suboptimal habitats where they eventually perish^21^. Moreover, invasive species inhabiting harbors are perfect model organisms to study present-day evolutionary processes following colonization, as most have repeatedly invaded multiple harbors worldwide over historical times^22^. Nevertheless, most invasive species lack a reference genome assembly properly annotated. The solitary tunicate *Styela plicata* is considered one of the most successful invasive marine species worldwide^23,24^. Although its native area is still unknown, it inhabits tropical and subtropical regions, thriving in artificial structures such as ports, aquaculture facilities, and ship hulls. It can reproduce throughout the year^25^, and their larvae have efficient mechanisms for settlement in artificial substrates, outcompeting other benthic species for space in early fouling stages^26^. An initial genetic study on *S. plicata* supported a worldwide panmictic population using the mitochondrial cytochrome oxidase 1 (*COX1*) and the nuclear adenine nucleotide translocase (*ANT*) markers^24^. However, a whole genome assessment on *S. plicata* is lacking and is necessary to fully understand sequence and structural variants to identify candidate adaptive regions and their potential functions.

**Fig. 1:**
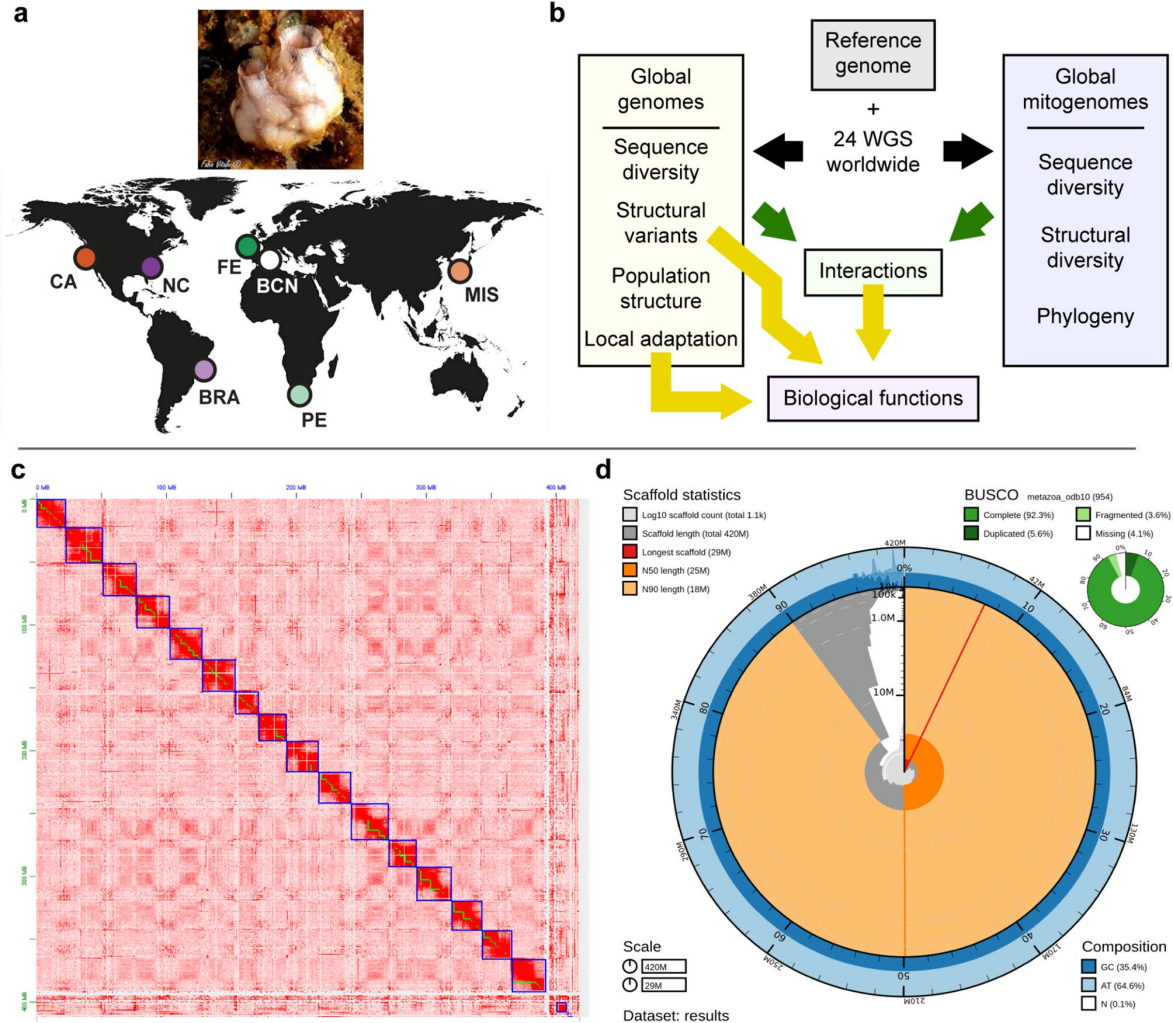
Sampling locations, analysis strategy, and reference genome features. (**a**): Sampling sites of *Styela plicata* individuals. An individual from Barcelona (BCN) was used for the construction of the reference genome. Individuals sampled in a previous work ^24^ from California (CA), North Carolina (NC), Santa Catarina (BRA), Ferrol (FE), Port Elizabeth (PE) and Misaki (MIS) were used for re-sequencing with short reads (WGS-SR). The map silhouette has been obtained with the R package ‘Rnaturalearth’ v.1.1.0 (https://CRAN.R-project.org/package=rnaturalearth). (**b**) Schematic flowchart followed in the present study. Black arrows indicate genome re-sequencing, green arrows the study of mitochondrial and nuclear interactions and yellow arrows the analyses to identify candidate genes for adaptation. (**c**) Omni-C contact map. Green and blue squares indicate contig and scaffold (combination of contigs) boundaries, respectively. (**d**) Snail plot of the final assembly and genome completeness based on metazoan BUSCO genes.

Here, we aim to generate the necessary genomic resources for *S. plicata* to understand the adaptive potential underlying the invasive success of this species in harbors. For this purpose, (1) we assembled and annotated the de novo chromosome-level reference genome of *S. plicata* and combined it with WGS data from 24 individuals sampled worldwide (Supplementary Table 1, Figure 1a). (2) We developed, validated, and applied iDlG (“individual Detection of linkage by Genotyping”), a novel user-friendly tool which allows the identification of multiple linked regions for several individuals simultaneously. (3) We detected population structure and adaptive candidate loci of the different genomic compartments considered. (4) We identified sequence and structural variants in the mitochondrial genomes, as well as nuclear genome regions associated with them. Finally, (5) we evaluated the biological functions of the genomic regions identified to be potentially driving *S. plicata* adaptation and resilience (Fig. 1b). By using this globally invasive species as a model, we aimed to stimulate future research on invasive species and to showcase the need to identify linked regions in the genome, thus taking a step forward in our understanding of species’ evolution and adaptation.

## Results

### Reference genome assembly and annotation

In total, 180 Gb were sequenced for PacBio CLR, 30.08 Gb for Illumina WGS-SR, 47.58 Gb for Illumina Omni-C, and 33.01 Gb for Illumina RNAseq (Supplementary Table 2). After filtering, we kept 46.17 Gb, 24.75 Gb, 45.12 Gb, and 16.08 Gb for PacBio CLR, WGS-SR, Omni-C, and RNAseq, respectively (Supplementary Table 2). The total length of the final assembly was 419.2 Mb (Supplementary Table 3), with a mean mapping coverage of 78X for PacBio CLR and 58X for Illumina Short Reads. The flow cytometry method provided a genome size estimation of 430 Mb, which was in line with the assembly results. The chromosome scaffolding (Fig. 1c) resulted in sixteen large scaffolds (398.9 Mb), in line with previously published karyotype data (n = 16)^27^. The Omni-C signal supported that most chromosomes were metacentric or submetacentric with similar sizes as described by Taylor (1967)^27^ (Fig. 1c). The genome assembly obtained for *S. plicata* is one of the highest quality in terms of NG50 (24,821,409 bp) compared to other tunicate assemblies (Supplementary Table 3). It had a QV statistic of 28.99, contained 90% of eukaryotic and 92.3% of metazoan single-copy BUSCOs (Fig. 1d, Supplementary Table 3), and most of the scaffolds were taxonomically assigned to Chordata (Supplementary Figure 2a). Albeit a chromosome had the best BLAST hit against Arthropoda, this is possibly due to a bias towards arthropods in public databases and the scarcity of ascidian sequences. Nevertheless, the GC content and coverage of that chromosome were consistent with those observed for all the chromosomes assigned to Chordata. The genome assembly of *S. plicata* was rich in repetitive elements (44.99%) including DNA transposons (4.22%), LINEs (4.11%), LTR elements (3.08%) and small RNA (2.07%), but also a large proportion of unknown repetitive families (35.7%) (Supplementary Figure 2b, Supplementary Table 4). Based on Kimura substitution levels of transposable elements (TE) families, *S. plicata* underwent an ancient TE expansion event mainly driven by unknown repetitive elements, DNA transposons, and a few LINEs. Similar patterns have also been reported for the sibling species *S. clava*^28^, suggesting that this expansion could have occurred in a common ancestor. Furthermore, and in contrast with *S. clava*, *S. plicata* suffered a second more recent TE expansion in which LTRs played an important role (Supplementary Figure 2c). After genome annotation, we recovered 37,097 genes, including 19,622 protein-coding genes, 7228 tRNA, 12,293 snoRNA, and 2451 lncRNA. In parallel, we successfully circularized the mitochondrial genome of the reference individual and obtained a unique contig of 14,413 bp with a mean coverage of 381X and a GC content of 29.9%. It comprised 13 protein-coding genes, two rRNAs organized in tandem, and 23 tRNAs (Supplementary Figure 3).

### WGS data curation and genomic diversity

On average, we sequenced ~ 35.90 million reads for each of the 24 individuals collected in six localities worldwide (Fig. 1a), from which ~ 35.85 million reads were kept after filtering (Supplementary Table 5). A mean of 97.8% of the reads per sample mapped to the reference genome, which represented ~ 11.8X mean coverage per sample (Supplementary Table 5). After SNP calling and filtering, we kept a total of 2,676,716 SNPs across all samples (Supplementary Figure 4a), showing fairly uniform nucleotide diversity (π) along the genome (Supplementary Figure 4b). Tajima’s D values were generally positive and high along the genome (from − 0.412 to 4.501, mean: 2.151, Supplementary Figure 4c).

### Chromosome inversion detection and functionality

**Fig. 2:**
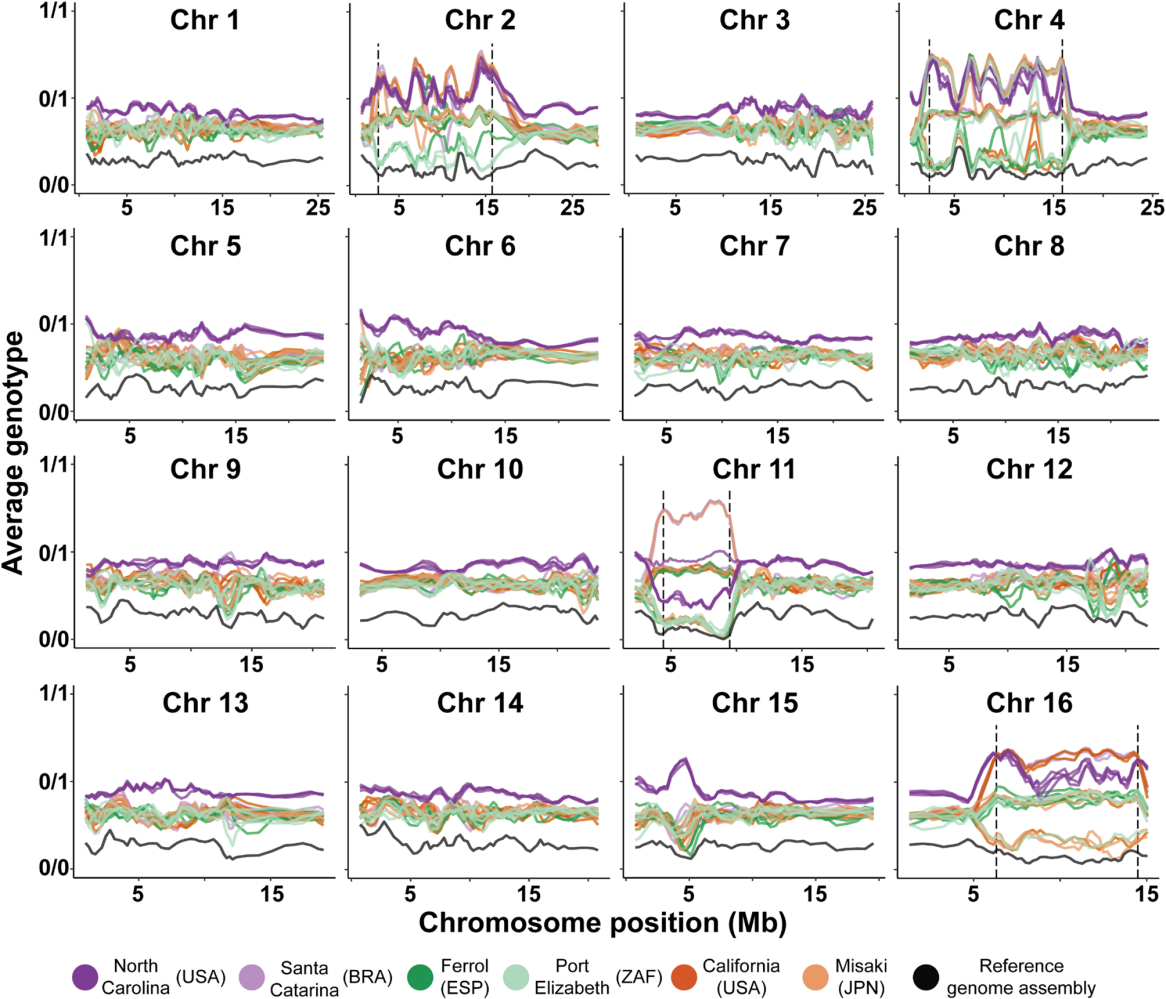
Average genotype for each individual across all chromosomes based on 10,000 SNPs windows with 2,500 SNPs sliding window inferred with iDlG. For each window the mean value is represented, and is calculated based on the recoded genotype where 0 indicates homozygous for the reference SNP (0/0), 1 indicates heterozygous (0/1), and 2 indicates homozygous for the alternative SNP (1/1). Note that homozygous blocks closer to 0/0 and to 1/1 identify individuals with homokaryotypes for reference and alternative linked regions in chromosome 2, 4 11 and 16. The short reads of the individual used to build the reference genome were also mapped and show no linked regions and its average genotype is close to 0/0.

**Fig. 3:**
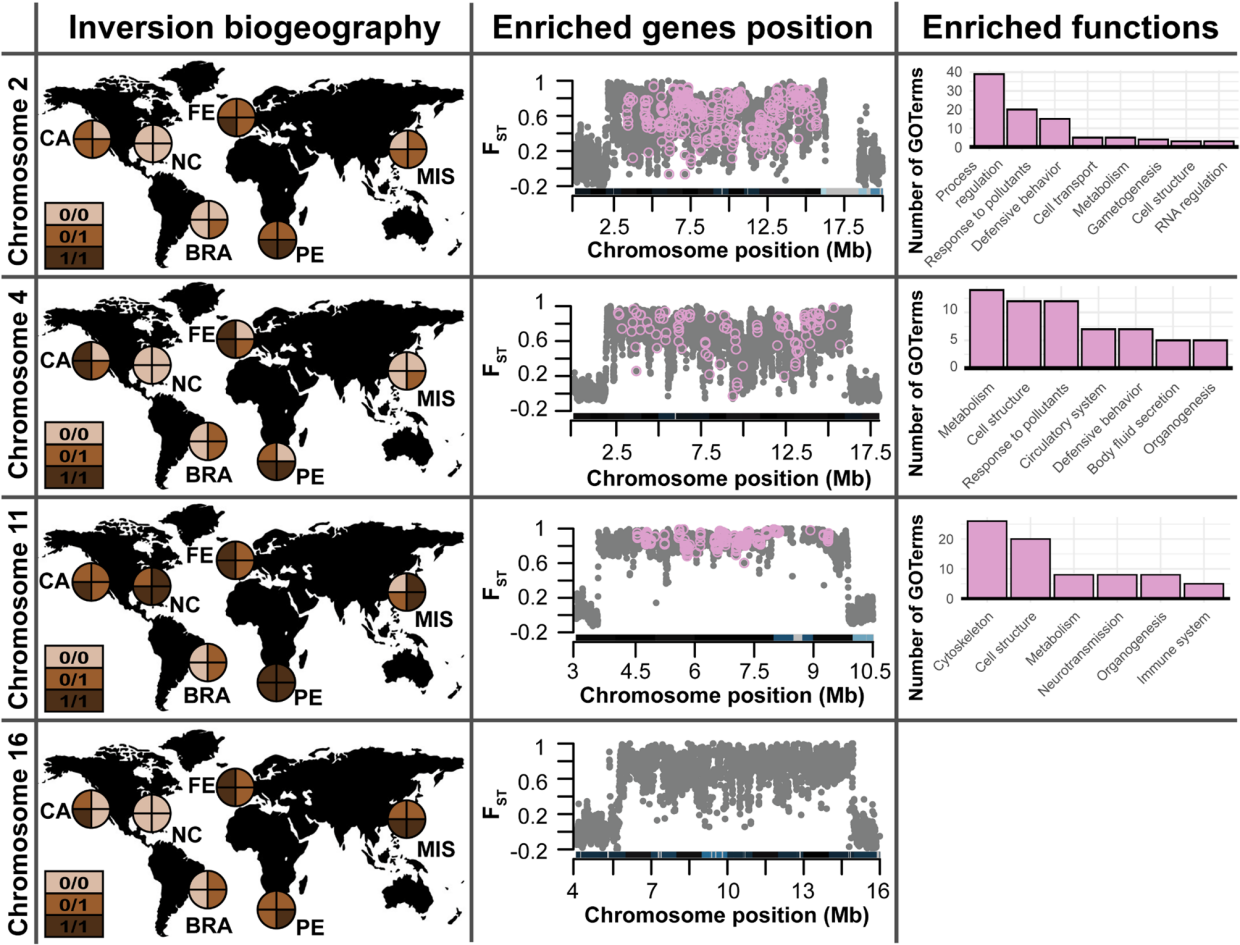
Distribution of chromosome inversions polymorphism and functional analysis. The first column shows the karyotype of the four individuals in each locality for each chromosome with identified inversions (0/0 = homokaryotype for the reference arrangement; 0/1 = heterokaryotype; 1/1 = homokaryotype for the alternative arrangement). Map silhouettes have been obtained with the R package ‘Rnaturalearth’ v.1.1.0 (https://CRAN.R-project.org/package=rnaturalearth). The second column displays Manhattan plots of the F_ST_ values between homokaryotype individuals identified with iDlG for each chromosomal inversion. Note that the region represented is a zoom-in of the linked region for better visualization of the shift in F_ST_ values. Light pink dots represent the locations of genes with enriched GO terms considering the area of influence detected by iDlG (Fig. 2, Supplementary Table 6). The third column provides the number of enriched GO terms belonging to the major clusters. Note that no enriched GO term was detected for the linked region in chromosome 16.

With iDlG, we identified large polymorphic linked regions in chromosomes 2, 4, 11, and 16, spanning 10.56% of the genome assembly. The presence of these linked regions, potentially chromosome inversions (see below), was detected as genomic blocks with divergent genotypes on these chromosomes (Fig. 2). For each chromosome, we defined the reference arrangement of the inversion as the haplotype whose SNPs were closer to the reference genome and the alternative arrangement as the haplotype with higher proportion of alternative SNPs. We defined the influence area of the inversions as the positions between the first and last maximum peaks of homozygosity (Fig. 2, Supplementary Table 6). This area represents the genome stretch where the two haplotypes are well differentiated, which we attribute it to the inversion span. Two peaks of high homozygosity were observed in chromosomes 11 and 16 (Fig. 2), with a plateau characterized by a long stretch of homozygous genotypes in between, with a slightly different pattern for individuals of North Carolina (NC, Fig. 2). Chromosomes 2 and 4 showed 4–6 internal peaks of homozygosity (Fig. 2). The analysis of the Illumina short reads from the reference individual against the assembled genome indicated that it was homokaryotypic for all detected inversions (Fig. 2). Among the 24 sequenced individuals worldwide, the inversions were polymorphic in most localities (meaning both chromosomal arrangements were present) with the exception of North Carolina, where only the alternative arrangement (in the opposite direction of the reference individual genome) was found in chromosomes 2, 4, and 16 (Fig. 3).

For the four chromosomes with potential inversions, the linkage disequilibrium (LD) approach recovered the same scenario of linkage as iDlG did. This method identified, for each chromosome with an inversion, a large region with correlation coefficients above 0.7 and an area of influence spanning a region similar to that found by iDlG (Supplementary Figure 5, Supplementary Table 6). The LD pattern found in chromosomes 2, 4, 11 and 16 was not observed in any other chromosome (Supplementary Figure 6). The local-MDS carried out on each of the four chromosomes with inversions, as identified with iDlG, confirmed their presence, visualized as three groups across the first MDS axis. These three groups reflected the three possible karyotypes for each inversion (homokaryotypes at both extremes of the MDS1 and heterokaryotypes in the middle). The karyotype detected for each individual matched the results obtained with iDlG (Supplementary Figure 7). The second MDS axis reflected genomic variation within chromosomal arrangements, possibly driven by the high divergence of NC (see Results). As found with the LD approach, the local-MDS pattern found in chromosomes 2, 4, 11 and 16, representative of chromosome inversions, was not observed in any of the other chromosomes (Supplementary Figure 8). In contrast, the first two axes of these MDS plots reflected a similar scenario of population structure, whereby NC was separated from all other localities in the first axis, and the latter clustered mostly according to their basin (Supplementary Figure 8). However, for some chromosomes without inversions individuals from BRA grouped closer to individuals from the Pacific (i.e. chromosome 8 and 9). Based on the karyotype obtained with both iDlG and local-MDS for each of the four inversions, we calculated F_ST_ values between the two groups of homokaryotype individuals for each chromosome. The results confirmed the presence of the inversions highlighted by iDlG, with shifts in F_ST_ values from 0 to 1 (Fig. 3b), mostly matching the area of influence detected with iDlG and the linkage disequilibrium analyses (Supplementary Table 6). Finally, we verified that the inversions in the four chromosomes showed a positive and significantly higher Tajima’s D values when compared to the values of all non-linked genomic regions (Wilcoxon test: *p* value < 0.001) (Supplementary Figure 9), indicating an effect of balancing selection.

To further evaluate the performance of iDlG in other species, we used a published WGS dataset of *Cyclopterus lumpus *^29^, a species that has an inversion reported on chromosome 2^30^. With iDlG, we successfully identified the influence area of the inversion (457,499 bp) as well as the karyotype of each individual (Supplementary Figure 10a). The finding with iDlG was again consistent with the patterns with linkage disequilibrium among the 64 individuals, the local MDS identifying the three karyotypes, and the F_ST_ analysis comparing only the two groups of homokaryotypes (Supplementary Figure 10b–d). The LD and the F_ST_ analyses indicated a similar region for the inversion as iDlG, and the karyotype of the 64 specimens were equally assigned with the local-MDS and iDlG. Thus, the validation using an independent dataset confirms that iDlG is a robust and reliable tool for detecting inversion in species other than *S. plicata*.

A comparison between the composition of genes found inside each *S. plicata* inversion according to the region of influence detected by iDlG relative to the rest of the genome, resulted in significantly enriched Gene Ontology (GO) terms in all four inversions except in chromosome 16 (Fig. 3, Supplementary spreadsheet 1). Interestingly, on each chromosome inversion, only five main functions encompassed more than 75% of the enriched functions (Fig. 3, Supplementary Spreadsheet 1). Moreover, the enriched functions were consistent regardless of the method (iDlG, LD, or F_ST_) used to define the area of influence (Supplementary Figure 11). When considering together all four inversions according to iDlG against all the genome regions outside the inversions, we detected an overall enrichment of genes related to response to pollutants, neurotransmission, and cell organization, among other functions previously recovered when evaluating each chromosomal inversion independently (Supplementary Figure 12, Supplementary Spreadsheet 1).

### Genetic differentiation and adaptation

**Fig. 4:**
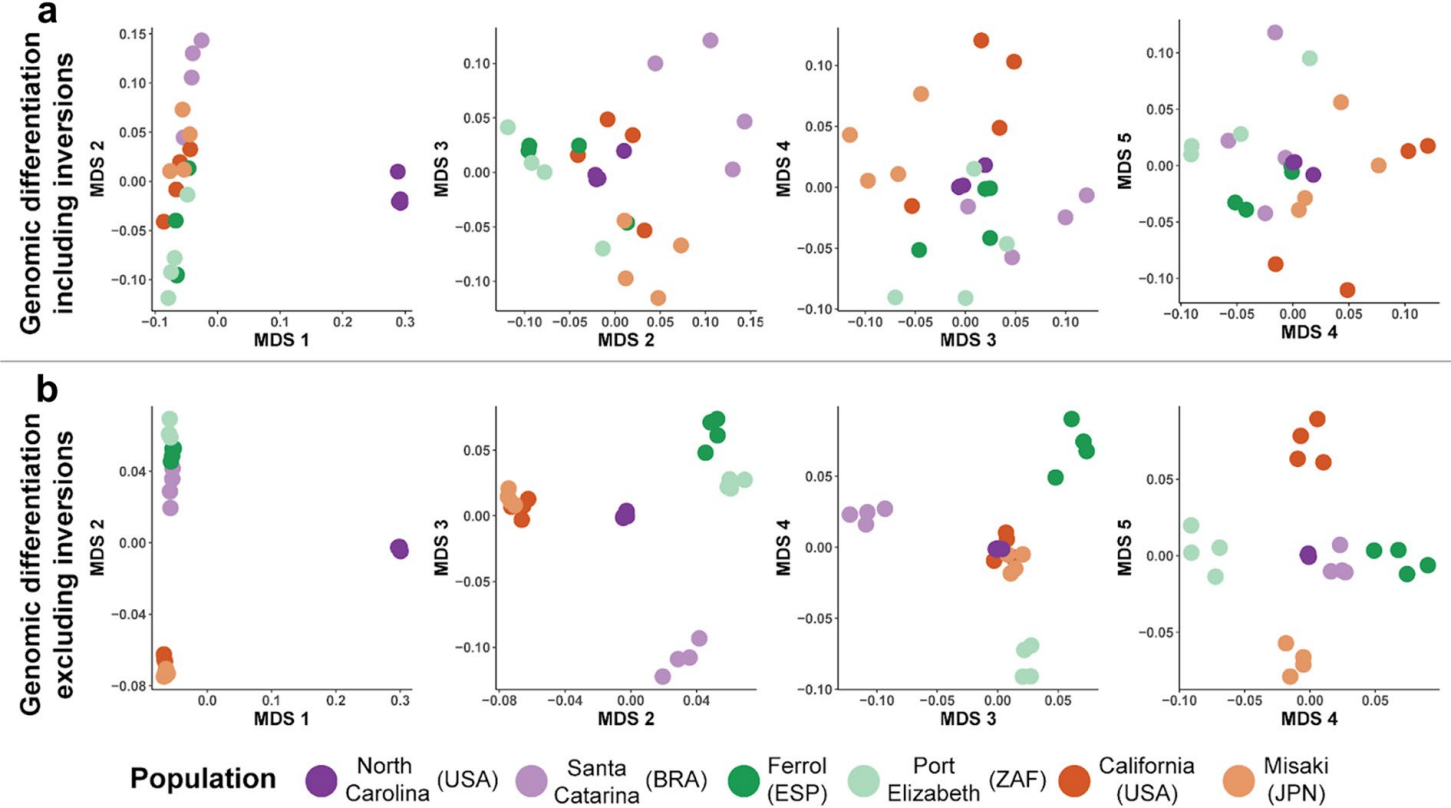
Genetic differentiation. (**a**) Genetic differentiation among individuals including all SNPs. MDS analysis plots for the five first axes, using all nuclear SNPs. Note that no clear population structure is recovered for any axis. (**b**) Genetic differentiation among individuals excluding SNPs in the four chromosomes with identified linked regions. MDS analysis plots for the five first axes using only the nuclear SNPs of the chromosomes without linked regions. Note that population structure is recovered hierarchically along the five first axes.

The genomic differentiation of *S. plicata*, obtained by including the chromosomes hosting inversions in the analysis, was inconsistent with the geographic distribution of the samples (e.g. samples from distant locations clustered together, while samples from the same locations appeared separated, Fig. 4a). Although the first axis separated North Carolina from all other localities, the other axes of the MDS failed to identify clear genetic differentiation between the remaining localities. However, when excluding the chromosomes containing inversions from the analysis, we recovered a clear geographic pattern of population structure along the first five axes of the MDS, with individuals from the same locality clustering together genetically (Fig. 4b). The five axes separated the individuals of the different localities hierarchically suggesting substructure within each oceanographic region. The first axis separated North Carolina from the rest, the second axis separated Pacific and Atlantic localities, the third axis West from East Atlantic localities, the fourth North and South East-Atlantic localities, and finally the fifth axis split the East and West Pacific localities (Fig. 4b).

Given the hierarchical biogeographic pattern of genetic differentiation found at the global scale of *S. plicata* when excluding chromosomes with inversions, we assessed F_ST_ patterns across the genome considering the groups obtained with the first and second MDS axes to test potential regional adaptation (Fig. 4b). According to the first axis, when comparing North Carolina with the remaining localities, we obtained very high F_ST_ values along the whole genome (Supplementary Figure 13), yet no outliers were found. In contrast, when considering the second axis, and thus the comparison between Atlantic (excluding North Carolina) and Pacific localities, although F_ST_ values were overall lower, potential signals of regional adaptation were found in the form of significant outlier F_ST_ values, detected with Rosner’s test (Supplementary Figure 14a). These outlier positions were found haphazardly distributed along the genome, although a region on chromosome 3 from position 26,182,501 to position 26,490,000 provided the strongest signal of potential regional adaptation between the Atlantic and the Pacific oceans (Supplementary Figure 14a). Genes included in this region were assigned to 49 GO terms (Supplementary Figure 14b). Metabolic processes, response to stimulus, peptidoglycan processes, behavior, and ion transport were the most represented functions.

### Mitogenome evolution and cyto-nuclear association

**Fig. 5:**
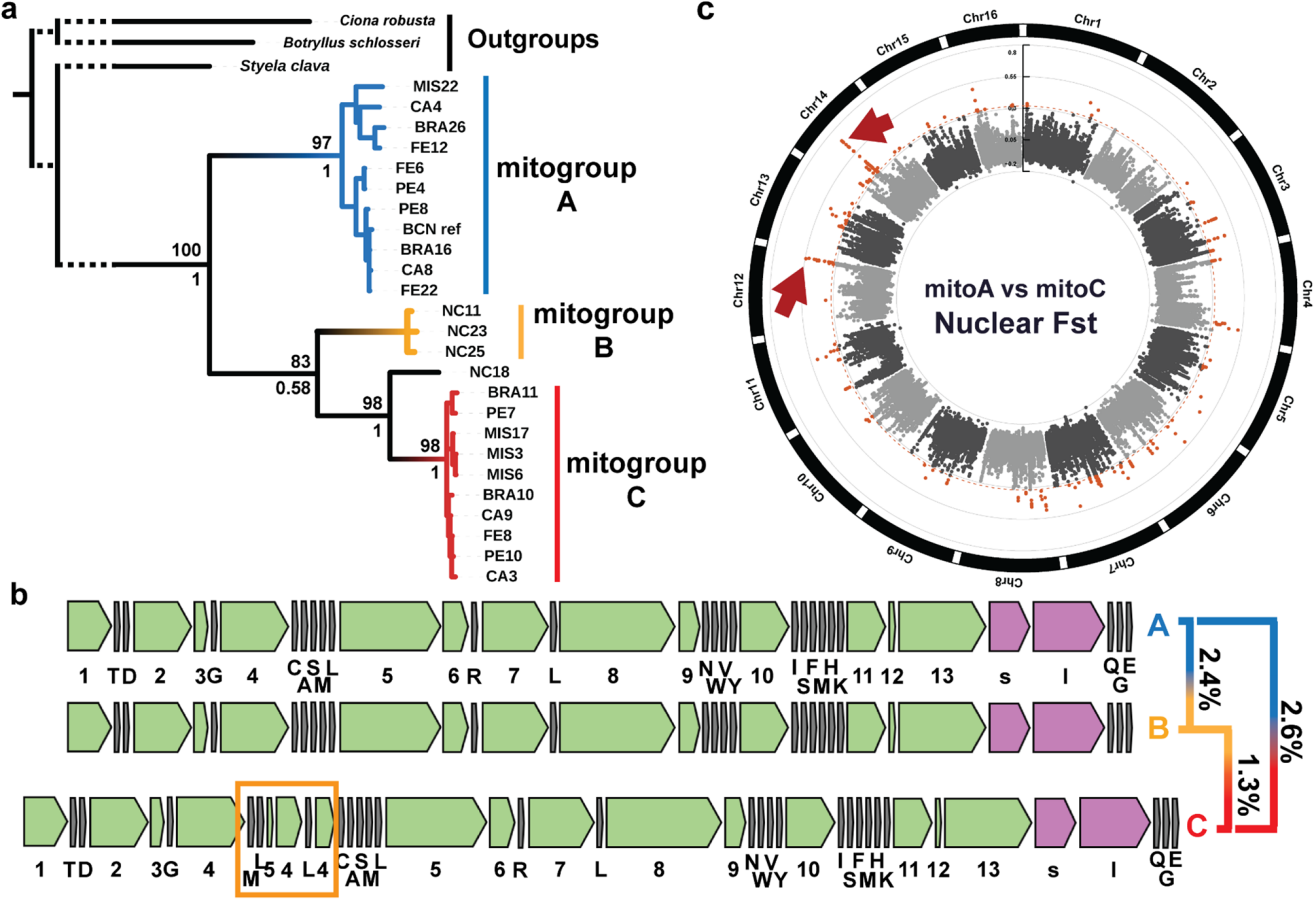
*Styela plicata* mitogenome diversity and divergence. (**a**) Maximum Likelihood phylogenetic tree inferred with the 24 individuals sequenced, using the mitogenome of *C. robusta*, *B. schlosseri*, and *S. clava* as outgroups. The tree topology belongs to the supermatrix approach. Supermatrix and supertree node support values are found above and below the branches, respectively. For visualization purposes, the tree is truncated in the root branches as denoted with a discontinuous line. Code alphabetic characters stand for NC = North Carolina, BRA = Santa Catarina, FE = Ferrol, PE = Port Elizabeth, CA = California, MIS = Misaki. (**b**) Mitogenome structural diversity and divergence. Mean mitogroup sequence divergence using the p-distance with Kimura-2 substitution model among the three major mitogenome clades. Numbers accompanying green boxes belong to coding genes, lowercase letters accompanying pink boxes belong to rRNA and capital letters accompanying gray boxes belong to tRNA and identify their associated amino acids (See code legends in “Supplementary Table 7”). Clade C has an insertion (orange rectangle) including two partial copies of *CYTB*, one partial copy of *COX1*, two additional tRNA-L and one additional tRNA-M. (**c**) Mitochondrial and nuclear genomes association. Nuclear F_ST_ values comparing individuals with mitogroups A and C in 10,000 bp windows with 2500 bp sliding windows. In orange, significant outlier F_ST_ values. Red arrows indicate regions used for the functional analyses.

All mitochondrial genomes were successfully circularized except for one individual from North Carolina (NC18). All orthologous genes were fully recovered in all individuals. Three main mitochondrial clades (mitogroups A, B and C) were inferred using both supermatrix and supertree approaches, with individual NC18 as sister of the mitogroup C clade (Fig. 5a). Regardless of the approach, mitogroup B was the sister clade to the NC18-mitoC group, and mitogroup A was the external clade. The mitogroup A clade matched those individuals identified as ‘haplogroup 1’ in previous studies with the cytochrome oxidase 1 gene^24^, while the former ‘haplogroup 2’ was split into mitogroups B and C. Mitogroup B encompassed only individuals from North Carolina, and mitogroup C included all individuals from the other localities of the former ‘haplogroup 2’ (Fig. 5a). These three mitogenome clades had structural and nucleotide variations (Fig. 5b). Mitogroup A was differentiated from mitogroups B and C by a mean genetic p-distance of 2.4% and 2.6%, respectively, while B and C had a p-distance of 1.3% (Fig. 5b). In terms of structural variation, mitogroup C was differentiated from mitogroups A and B by a large insertion of approximately 1,000 bp, which included two partial copies of the gene cytochrome oxidase b, one partial copy of the gene cytochrome oxidase 1, and three full tRNA copies (Fig. 5b). The insertion was confirmed when the reads of each mitogenome were mapped against the circularized sequences of the three mitogroups (Supplementary Figure 15). Interestingly, the reads of individual NC18— the only mitochondrial genome that could not be circularized—indicated that most of the insertion of mitogroup C is also present in NC18, although with some deletions and additional duplications (Supplementary Figure 15).

Mitogroups A and C were found in sympatry across all populations with the exception of NC (Fig. 5a). In order to detect nuclear regions associated with the two mitochondrial groups and avoid biases due to population differentiation, we performed nuclear F_ST_ analyses comparing individuals from localities with an equal number of representatives with mitogroups A and C (CA, PE, BRA). We found a few nuclear markers differentiating the two mitogroups (A and C) according to the F_ST_ outlier analysis (outliers detected with Rosner’s test), mostly scattered along the genome but densely aggregated in two regions of chromosomes 12 (21,797,501–21,870,000 bp) and 14 (8,762,501–8,797,500 bp) (Fig. 5c). Overall, the biological function of the genes in these two regions was not involved in cellular respiration, but in regulation processes, metabolism, RNA and protein editing, and development (Supplementary Figure 16, Supplementary Spreadsheet 1). The cellular functions of the genes within these regions were related to Sm-like proteins, the cytoskeleton, and membrane transport (Supplementary Figure 16, Supplementary Spreadsheet 1). For chromosome 12, the genes located in the window with highest F_ST_ (21,830,001–21,840,000 bp) provided no BLAST matches. The BLAST of the window with the highest F_ST_ of chromosome 14 (8,772,501–8,782,500 bp), identified the gene ‘UDP-galactose/UDP-glucose transporter 7-like’.

## Discussion

### Beyond a single nuclear reference genome

The generation of reference genomes has become a milestone in genomics^8^. Here, we have generated the reference genome of the invasive ascidian *Styela plicata*, a model system to study invasion patterns given its present-day global distribution. The quality of this genome stands out in comparison with other ascidian species. *S. plicata* is one of the few tunicate species whose genome has been assembled to the chromosome level and has an associated annotation. However, the more we advance in the knowledge of genomes of living organisms, the more we understand their complexity and the importance of analyzing multiple genomes within species to capture the species-specific diversity^12,31^. The combination of *S. plicata*’s reference genome assembly with whole genome sequencing data from 24 individuals across its whole distribution range to generate a catalog of structural and sequence variants has further enhanced our knowledge on this species. This newly generated genomic resource has been paramount to assess the evolutionary history of the species, using a reduced representation approach^32^. Our study has shown that the genome of this species cannot be considered as a single unit, but as a compendium of different regions which reflect different evolutionary signals that interact to produce a diversity of phenotypes. This is because different genome regions (both nuclear and mitochondrial) have been shaped by different evolutionary forces, with potential interactions between different genomic compartments. Consequently, by dissecting the different components of the genome and analyzing them independently, we have been able to ascertain multiple signals of adaptation that may enhance the invasive success of this species worldwide.

### iDlG, a new R script for identifying linked regions

The identification of inversions and other linked regions has become an important focus of current genomic research. These regions are important to characterize since, as observed with the present dataset, diverging signals can have an overwhelming effect on subsequent analyses, blurring population structuring and adaptation signals. Large linked regions are related to structural chromosomal variation and, among them, chromosomal inversions are the most common and have been demonstrated to play key roles in adaptive evolution^14^. Inversions are often detected by comparing the genomic data of well-defined discrete morphotypes or ecotypes through sliding-windows F_ST_ analyses, which return regions with extremely high values of differentiation^18^. However, many species do not have morphological or ecological variation to start with. This is the case for *S. plicata* individuals, which do not have discrete morphotypes or ecotypes (pers. obs.), making it impossible to use this methodological approach.

To overcome this limitation, we have developed iDlG, a new method that employs genotype sliding windows to simultaneously detect multiple chromosomal linked regions, their area of influence, and the karyotype of each individual without requiring previous morphological or ecological information. By using iDlG and 25 genomes of *S. plicata* across its distribution range (including the individual of the reference genome), we have found four large regions with linked genotypes. These regions most likely correspond to chromosome inversions, as large non-recombining areas require physical hindrances to avoid recombination and to be maintained over generations^33^. Importantly, the iDlG results were in line with the LD, F_ST_, MDS, and Tajima’s D analyses. Additionally, the iDlG method could also recover the previously reported inversion in chromosome 2 of *Cyclopterus lumpus*. Thus, our study validates iDlG, and demonstrates its applicability on any WGS dataset regardless of the species. Chromosomal inversions are known to reduce recombination between chromosomes in heterokaryotypes^33^, therefore promoting the differentiation of the haplotypes in the inverted region, as observed by both iDlG and F_ST_. However, low recombination rates in heterokaryotypes can extend beyond the potential inversion breakpoints as identified with iDlG, making it difficult to precisely identify inversion haplotype boundaries^14^. Finally, as iDlG shows the basal average genotype per window of each individual, we could identify that samples from NC consistently present higher basal values across all regions of the genome except within linkage regions, reflecting population-specific genomic patterns. Thus, the potential of iDlG can go beyond inversion and other linkage region discovery, also shedding light on the basal genome-wide differentiation of specimens included in the analysis and providing evidence on population differential patterns.

We encourage the use of iDlG in future population genomic studies using whole genome re-sequencing techniques with short reads, as well as with long reads, for a fast identification and assignation of structural variants without requiring multiple reference genome assemblies, and for obtaining individual-level differentiation values which can reflect locality-specific patterns. Structural variants such as inversions should be investigated in all species across the Tree of Life since they can provide valuable clues to the potential for adaptation and colonization in the context of the current drastic global environmental changes^14^. At the same time, including inversions in the analyses can mask population differentiation, as found in the present work, thus hindering the assessment of adaptation processes during colonization of new areas.

### Structural variants role on worldwide invasive success

Chromosome inversions have an adaptive role by maintaining co-adaptive gene complexes^34,35^. The maintenance of different inversion arrangements within populations has been reported to be instrumental in survival to harsh environmental changes^35,36^. The rich inversion polymorphism found in *S. plicata* and its worldwide distribution suggest that they could be adaptive and have implications for its invasive capabilities, as found in other species^36^. In North Carolina, the fixation of almost all inversions (except for chromosome 11) may indicate different evolutionary processes in this locality, in line with results obtained using reduced representation genomic data^32^. North Carolina is characterized by fixed inversions and an overall high genetic differentiation when compared with all other samples, which might indicate that this locality is an isolated population. In other invasive tunicates, it has been described that some genotypes have invasive potential while others do not^37,38^, which could also be the case for *S. plicata*, specifically the NC locality. In any case, the presence of polymorphic inversions in all localities but NC suggests that *S. plicata* may benefit from these inversions to spread worldwide. Further research including a wider taxon sampling should be done to assess karyotype frequencies across localities and verify the role of inversions in the invasive process. The NC population also deserves further studies to unveil its relationship with the expansion of the species due to the high differentiation levels at the nuclear genome relative to the other localities and their exclusivity of mitogroup B lineage.

It is worth noting that, in *S. plicata,* each chromosomal inversion is enriched in genes for different biological processes, suggesting that each inversion may play a specific role in the species’ success. Most of these functions can be associated with fitness enhancers in estuarine and harbor environments, the habitat where *S. plicata* thrives, such as the immune system (chromosomes 11 and 16)^39^, reproduction (chromosomes 2 and 16)^40^, response to pollutants and other stressors (chromosomes 2, 4 and 16)^41,42^, or cell division and cellular organization processes (chromosome 11), among others. The concentration of enriched functions in inversions suggests that inversions might be relevant for adaptation. Consequently, inversions maintained worldwide in a polymorphic state could act as standing variation to cope with environmental shifts^14^. This process has been extensively studied in *Drosophila subobscura*, where the polymorphism of multiple inversions including genes related to thermal stress is maintained across localities and differentially selected to cope with colder and warmer climates, climate seasonality, and heatwaves^43–45^. In *S. plicata*, the presence of polymorphic inversions with genes related with immune system and response to pollutants might allow this species to overcome a wide arrange of different polluted environments, such as harbors, when colonizing worldwide. Nevertheless, future experimental studies exposing individuals with different karyotypes to different conditions are necessary to fully understand the functional and adaptive role of these inversions.

### Population structure and adaptation

A correct assessment of genetic differentiation using population genomics is a powerful tool for proper management and conservation. Nonetheless, using genomic resources does not guarantee recovering reliable population differentiation. Our population study analysis has evidenced that chromosomal inversions can mask population structure. The alleles found within inversion variants can remain mostly unchanged across large geographic regions^46^. Consequently, the co-occurrence of multiple divergent chromosome inversions within localities could likely mask the genomic differentiation among *S. plicata* individuals when including whole genome data. Upon removal of the inversions, we have uncovered a clear population structure for this species, with a separation of the Atlantic and the Pacific oceans. The same pattern in *S. plicata* was also observed when considering or removing the four chromosomal inversions characterized in the present work but using 2b-RAD approaches and a larger number of localities^32^. However, our results show that using whole genome sequencing data improves population differentiation detection since we clearly recover all localities as independent genomic units across the MDS axes. This is because with WGS, the number of SNPs obtained is three orders of magnitude higher than with 2b-RAD. Thus, our results indicate that the degree of population structuring of *S. plicata* is stronger than previously thought. Since *S. plicata* is an invasive species thought to have colonized oceans worldwide during the last three centuries, this may indicate that *S. plicata* experienced many locality-specific evolutionary changes by both genetic drift or natural selection and adaptation.

Adaptation at the local scale is crucial for species survival, distribution range expansions and, especially, during invasion processes. This is because invasive species can reach distant habitats, facing new conditions that might compromise the survival of colonizer specimens. We have found that one of the main functions of candidate loci for adaptation implicated in geographic differentiation was related to ion transport. Given the fact that the Atlantic Ocean salinity is slightly higher than the Pacific, individuals from both areas may need to osmoregulate differently in each ocean, driving local adaptation, as similarly reported in fish or sea urchins^47,48^.

Overall, our data demonstrate that the genome encompasses different regions that may be subject to different constraints and thus reflect different evolutionary processes. Consequently, it is crucial to uncover in advance sequence and structural variants, such as chromosomal inversions and other linked regions, to fully capture the potential of genomics in population studies. We encourage future genomic studies on any species to initially test for potential structural features indicated by divergent genotypic blocks with the help of reference genomes. This can only be done by assessing genome diversity by analyzing WGS data from multiple specimens of the same species to uncover hidden genomic features and their importance for correct and reliable interpretations of the results of genomics analyses^31,49^.

### Evolutionary signals of mitochondrial lineages

The inclusion of both mitochondrial and nuclear data is of utmost interest for genomic studies, as analyses of both types of molecular marker can provide a more complete picture of the species’ evolution due to the general lack of recombination in mtDNA and differences in mutation rates between the two genomic compartments^50^. As found in previous studies^24^, the differentiation of mitochondrial DNA of *S. plicata* does not match its geographical distribution, as individuals from the two more differentiated and abundant clades (A and C) are globally sympatric. The fact that the two most divergent mitogenomic clades are present in almost all localities suggests that these two clades evolved in allopatry and that subsequent secondary contact occurred before the worldwide expansion of the ascidian, homogenizing nuclear markers due to recombination, whereas mitochondrial genomes remained distinct because they are maternally inherited and do not recombine. Mitogroup B, on the other hand, is present only in individuals from North Carolina. Although the NC population might look like a different species based on nuclear markers, the phylogenetic position of the mitogroup B lineage between mitogroup A and mitogroup C clades rejects the species complex hypothesis, confirming the identification of all specimens to *S. plicata* as correct.

Additionally, evaluating the functionality of the regions of the nuclear genome associated with the different mitochondrial lineages is of great importance to understand the combined role of both mitochondrial and nuclear genomic compartments on adaptation. Our F_ST_ analyses comparing the mitogroups A and C showed high values of nuclear divergence associated with the mitochondrial group, concentrated mostly on localized regions of chromosomes 12 and 14. This signal provides evidence of associations and possible interactions between these two genetic compartments. These interactions are not surprising, since the mitochondrial and the nuclear genome are known to coevolve to maintain overall functionality and, consequently, the viability of individuals^51,52^. Our analyses on chromosome 14 found a BLAST match with a ‘UDP-galactose/UDP-glucose transporter 7-like’ gene. This gene is involved in the correct transport of galactose and glucose to the Golgi vesicles^53^, which promotes glycosylation of several proteins to enhance their performance. Some studies have indicated that glycosylated proteins might play important roles in mitochondria by modifying and regulating the functions of the non-glycosylated original isoforms^54^. On the other hand, the GO term analysis found Sm-like proteins in chromosome 12, which are described to play important roles in alternative splicing^55^. Interestingly, Sm-like proteins of chromosome 12 could be associated with the UDP-galactose/UDP-glucose transporter 7-like of chromosome 14, as Sm-like protein activity occurs inside the Golgi vesicles. Golgi elements are tightly associated with mitochondria in many animals during oocyte development, generating a structure named the Balbiani body. This structure is associated with ribonucleoproteins with a core domain ruled by Sm-proteins^56,57^. Thus, in the context of potential coevolution between mitochondrial and nuclear genes of *S. plicata*, organelle organization and the synthesis of protein isoforms might play an important role in reproductive compatibility of the invasive tunicate *S. plicata* that should be further evaluated. As whole genome sequencing is being increasingly used for population genomics worldwide, we encourage the use of our approach to explore potential associations between mitochondrial and nuclear genomes, to reliably identify coadaptation mechanisms between these two cellular compartments across species.

## Conclusions

Our work with the invasive species *Styela plicata* has showcased that a reference genome and re-sequencing data of multiple individuals across the species distribution is fundamental to capture genomic diversity and, when possible, we advocate for implementing this procedure in population and conservation genomics. We have developed iDlG, a new tool to identify linked regions based only on genomic data, without the need for prior knowledge, and have demonstrated that detecting chromosomal inversions in advance can be crucial for uncovering multiple nuclear genomic compartments shaping the species genetic differentiation and adaptation. Moreover, we show that, if genome structural diversity is not considered, partial or spurious results may be obtained, by mixing confounding signals from different genomic regions. Thus, by identifying different genomic compartments, we could evaluate their diversity, as well as their potential interactions and role in adaptation. We found that chromosomal inversions in *Styela plicata* are polymorphic and enriched with genes linked to adaptation in harbors, but mask population structure that becomes clear only after their removal. We also identified three mitogenomic lineages, some associated with nuclear genes involved in mitochondrial distribution, highlighting the importance of integrating nuclear and mitochondrial data to understand complex evolutionary processes. Overall, we strongly emphasize the need of intraspecific genomic studies, the next logical step after building reference genomes, allowing for a comprehensive understanding of the processes underlying population evolution.

## Material and methods

### Reference genome sampling and sequencing

Two *Styela plicata* individuals were collected in the harbor of Barcelona in September 2020 (Supplementary Table 1, Fig. 1). One of the individuals was kept alive until DNA extraction to best preserve DNA integrity, while the other was immediately preserved in RNAlater for RNA extractions. Once in the laboratory, two gill folds were excised for DNA extraction and genome size estimation, while 25 mm^2^ each of gill, mantle, and gonad tissues were selected for RNA extractions.

DNA extraction followed a protocol based on that of Mayjonade et al. (2016)^58^. RNA extractions were performed according to the protocol of Ghangal et al. (2009)^59^. The quality and concentration of both extractions were assessed using the TapeStation 2200 (Agilent Technologies) and a Qubit Fluorometer device with the appropriate Qubit dsDNA/RNA BR Reagents Assay Kit (Thermo Fisher Scientific, Waltham, MA).

A SMRTbell library was constructed following the instructions of the SMRTbell Express Prep kit v2.0 with Low DNA Input Protocol (Pacific Biosciences, Menlo Park, CA). One SMRT cell sequencing run was performed in CLR mode on a Sequel System II with the Sequel II Sequencing Kit 2.0. Additionally, a DNA extract of the same specimen was sent for Illumina Whole Genome Sequencing Short Reads (WGS-SR) to Novogene (UK) Co. Ltd. One genomic library (insert size: 350 bp) was prepared, and 150 bp paired-end reads were sequenced on an Illumina NovaSeq 6000 platform (San Diego, CA) targeting 30 Gb output (~ 60 × coverage according to an estimated genome size of 430 Mb; see Results). Omni-C short reads were obtained by building the corresponding libraries following Dovetail^Ⓡ^ Omni-C kit manufacturer’s instructions with an insert size of 350 bp and using a 150 bp paired-end sequencing strategy as for WGS-SR. Finally, the RNA extractions from gill, mantle and gonad tissues were pooled at equal concentrations and sent to Novogene (UK) Co. Ltd. for a cDNA library construction (insert size: 350 bp) and sequencing of Illumina paired-end 150 bp RNA-seq with an expected output of 30 Gb on the same platform (Supplementary Table 2).

### Genome size estimation

Genome size was estimated following a flow cytometry protocol with propidium iodide-stained nuclei described in Chueca et al. 2021^60^. Fresh mantle and gill tissues of *S. plicata* were separately chopped with a razor blade in independent Petri dishes containing 2 ml of ice-cold Galbraith and Otto buffer, respectively^61,62^. As internal reference standards we used female cricket head tissue (*Acheta domesticus*, 1C = 2 Gb) and commercial chicken nuclei (*Gallus gallus*, 1C = 1.2 Gb) (Biosure). The suspension was filtered through a 42 μm nylon mesh and stained with the intercalating fluorochrome propidium iodide (PI, Thermo Fisher Scientific) and treated with RNase II A (Sigma-Aldrich), each with a final concentration of 25 μg/ml. The mean red PI fluorescence signal of stained nuclei was quantified using a Beckman-Coulter CytoFLEX flow cytometer with a solid-state laser emitting at 488 nm. Fluorescence intensities of 5000 nuclei per sample were recorded. The software CytExpert 2.3 was used for histogram analyses. The total quantity of DNA in the sample was calculated as the ratio of the mean red fluorescence signal of the 2C peak of the stained nuclei of the *S. plicata* sample divided by the mean fluorescence signal of the 2C peak of both reference standards. Three replicates for *S. plicata*, *A. domesticus*, and *G. gallus* were measured on three different days to minimize instrument error.

### Reference genome assembly

PacBio CLR subreads raw sequence files (BioProject PRJEB67507) were transformed from bam to fastq format using the command ‘fastq’ from samtools v.1.10^63^ and their quality was assessed with Nanoplot v.1.28.1^64^. Nanofilt v.2.6^64^ was used to apply a minimum length threshold filter of 25 kb (-l 25,000) to remove potential bacterial contamination and mitogenomic sequences shown as secondary GC content peaks in fastQC plots (Supplementary Figure 1). Both Omni-C and WGS-SR data (BioProject PRJEB67507) were trimmed and filtered using Trimmomatic v.0.39^65^ using the following parameters: ILLUMINACLIP:TruSeq3-PE.fa:2:30:10 LEADING:3 TRAILING:3 SLIDINGWINDOW:4:15 MINLEN:36, and the resulting filtered reads quality was measured with fastQC v.0.11.9^66^.

We used Flye v.2.8^67^ to assemble the filtered PacBio CLR data predefining a genome size of 430 Mb (-g 430 m, see Results), a minimum overlap between reads of 10,000 bp (-min-overlap 10,000), and three iterations of self-polishing (-i 3). We then conducted three polishing rounds with WGS-SR data using Pilon v.1.23^68^ using default parameters. Finally, the assembly draft was deduplicated using purge_dups.py v.1.2.6^69^ using default parameters. For genome scaffolding we used Omni-C data together with the polished deduplicated assembly in the program Juicer v.1.6^70^, jointly with a cut-site position file previously generated with the script of the same program generate_site_positions.py defining ‘none’ as the restriction enzyme, as Omni-C libraries use an endonuclease with random cleavage sites. The function run-asm-pipeline.sh of the program 3d-dna v.201008^71^ was called to obtain the contact map, specifying a haploid sequence (-m haploid), a minimum contig size to be considered of 1000 bp (-i 1000), and four rounds of iterative scaffolding correction (-r 4). The resulting scaffold map was manually curated using the interface of Juicebox v.1.5^72^, and the final chromosome-level assembly was recovered by running the script juicebox_assembly_converter.py of the same program.

Coverage along the genome assembly and mean coverage were tested by mapping the initial CLR and WGS-SR back to the polished assembly using the default parameters of the software Minimap2 v.2.24 and BWA-mem v.0.7.17, respectively, and visualized with Qualimap v.2.2.1 and multiQC v.1.8^73–76^ implemented in backmap.pl v.0.4^77^ https://github.com/schellt/backmap. Contiguity statistics were obtained using QUAST, base-level accuracy (qv) was assessed with mercury v.1.3^78^, and genome completeness was corroborated by detecting universal single copy orthologs of metazoa (metazoa_odb10) using BUSCO v.5.2.2 (Wright, 2012). We checked for DNA contaminations by comparing our sequences to those in NCBI’s nt database using BLAST v.2.12^79^, with the algorithm (-task) megablast, retrieving 10 hits per sequence (-max_target_seqs 10), a minimum e-value of 1 × 10^−25^ (-evalue 1e−25), and printed the hit with highest High-scoring Segment Pairs (-max_hsps 1). The resulting quality features values were graphically represented using the software BlobTools v.2.0^80^. As a result, we generated a high-quality chromosome-level reference genome assembly for subsequent analyses. The reference genome assembly was uploaded to ENA as BioProject PRJEB67507.

### Reference genome annotation

We downloaded from repbase^81^ previously reported transposable elements (TE) of the model species *Ciona intestinalis* type A (*Ciona robusta*), since it was the species most closely related to *S. plicata* with available TE data. Furthermore, we also downloaded the reference genome of the congeneric species *Styela clava*^28^ to aid in our TE annotation. We used default parameters on RepeatModeler v.2.0^82^ in both *S. plicata* and *S. clava* to generate de novo predictive TE models for the genus *Styela*. The resulting models obtained for both species were combined with the TEs of *C. robusta*. The model file including the TEs of all three species was used to soft- (-xsmall) and hard-mask the chromosome-level assembly with RepeatMasker v.4.1.2^83^. We plotted each TE family abundance and Kimura2 substitution levels profile using the program RepeatLandscape.pl, included in RepeatMasker, setting an assembled genome size of 420 Mb (see Results).

For gene annotation, we downloaded a protein set of Stolidobranchia from UniProt^84^. In addition, RNA-seq data from gill, mantle, and gonads (BioProject PRJEB67507) previously filtered with Trimmomatic v.0.39 was assembled into transcripts with Trinity v.2.11^85^ using default parameters. To reduce redundancy, we merged and clustered all transcripts with more than 99% similarity (-c 0.99) using CD-HIT v.4.8^86^. Both the Stolidobranchia protein set and our RNA-seq data were used to annotate the hardmasked genome using BLAST and exonerate, both implemented in MAKER v.2.31.10^87^. Gene *ab-initio* predictions were conducted by AUGUSTUS v.3.4.0, GeneMark-EP v.4.71 and SNAP v.2013-11-29, as implemented in MAKER^88–90^. Three rounds of protein modeling were carried out to refine the genome annotation. For the first modeling round, complete BUSCO genes (metazoa_odb10) were used to generate a gene model with SNAP, whereas AUGUSTUS and GeneMark were based on RNAseq. After the first modeling, an annotation draft was obtained with MAKER. For AUGUSTUS and SNAP, second and third model rounds were carried out using as a training set those genes obtained by the most updated annotation draft. The new training models were used to reannotate the genome assembly. Additionally, in the last annotation round, tRNAscan-SE v.2.0^91^ and snoscan v.0.9.1^92^ were activated in MAKER to annotate tRNA and small non-coding RNA (snoRNA). As the last step, we predicted long non-coding RNA (lncRNA) with FEELnc v.0.0.1^93^ and CPC2^94^ using default parameters. lncRNA shared between both softwares not overlapping with any protein coding gene were selected as reliable lncRNA candidates and ultimately included in the *S. plicata* annotation^95^. Finally, genes were compared against Pfam databases using eggNOG-mapper v.2^96^ with default thresholds in order to assign Gene Ontology terms (GO terms), and the best match was recorded in the final annotation file.

### Worldwide genome sequencing and genotyping

To assess the genome variation worldwide, we sequenced 24 additional *S. plicata* individuals from different harbors previously studied in Pineda et al. (2011)^24^: California, USA (n = 4), Santa Catarina, Brazil (n = 4), North Carolina, USA (n = 4), Ferrol, Spain (n = 4), Port Elizabeth, South Africa (n = 4), and Misaki, Japan (n = 4) (Supplementary Table 1, Figure 1a). Since the cytochrome oxidase I (*COXI*) haplotypes of these samples were already known^24^, we chose whenever possible a balanced proportion of individuals with the two described haplogroups in every locality to evaluate whether there are nuclear regions associated with the two mitochondrial groups and avoid population biases (Supplementary Table 1). DNA extractions were sent to Novogene for WGS-SR library preparation and sequencing of 5 Gb 150 bp paired end reads, aiming for an approximate coverage of 12X per individual.

Illumina WGS-SR data of the 24 worldwide sampled individuals were filtered with Trimmomatic v.0.39 and deposited in ENA (BioProject PRJEB67519). Filtered WGS-SR reads of these individuals and of the individual used to obtain the reference genome were mapped to the newly generated chromosome-level reference genome using BWA-mem, and genotypes were called using the “mpileup” function in BCFtools v.1.10.2^63^. VCFtools v.4.2 was used for filtering variants^97^. We retained those biallelic SNPs with a minimum coverage of 5 reads (-minDP 5) and removed loci with a mean coverage across samples above 25 reads (-max-meanDP 25). This threshold was calculated as 1.5 times the interquartile range for loci mean coverage. Next, we applied a minimum allele frequency filter of 10% (-maf 0.1) and only kept those SNPs present in 100% of the individuals (-max-missing 1).

### Identification of intra-chromosomal linkage with iDlG

We developed a method called “individual Detection of linkage by Genotyping” (iDlG), implemented in an in-house R script (https://github.com/CGaliaCamps/iDlG), This approach assumes that recombining genomic regions share a baseline level of heterozygosity across all individuals of a given species population, although this baseline may vary among different chromosomes and even within the same chromosome since recombination varies across them^98^. In contrast, non-recombining regions are expected to reflect a substantial deviation from this baseline, showing signals of fixation either towards the reference (the SNP present in the reference genome) or the alternative arrangement. Based on this rationale, iDlG is an easy-to-use tool to detect and visualize linked blocks along the chromosomes at the individual level and their potential area of influence without previous information of the genetic linkage or of phenotypic/ecologic differentiation. The detection of linkage is based uniquely on the SNP genotype across the chromosome recodified in the 0/1/2 format using the function -012 from VCFtools v.4.2, where 0 indicates homozygous for the reference SNP (the same allele as the reference genome), 1 heterozygous, and 2 homozygous for the alternative SNP. The script calculates the mean value of the genotypes in SNPs windows defined by the user. To execute the script, iDlG requires specification of two primary parameters: “window” and “step.” The window parameter defines the number of SNPs included in each segment used to calculate the mean genotype, whereas the step parameter specifies the number of SNPs the window advances at each iteration. Importantly, window size should be calibrated to the SNP density of the dataset. High SNP-density datasets will allow the use of both large and small windows, whereas low SNP-density datasets will require only small windows to capture linkage blocks. On high SNP-density datasets, selecting a small window size enhances the ability to detect fine-scale linkage blocks or micro-inversions. In contrast, a large window size reduces sensitivity to identify small linkage regions. Once the mean genotype for each window has been computed, iDlG plots the results for each individual using the R package ‘ggplot2’ v.3.4.1^99^. Putative different chromosomal inversions can be identified as blocks with more homozygous genotypes (closer to 0 or to 2) than the baseline heterozygosity signal, a pattern consistent with suppressed recombination and fixation of alternative alleles.

For the present dataset, with the aim of detecting macro-inversions at a low computing cost, we used overlapping sliding windows of 10,000 SNPs (window = 10,000) with 2,500 SNPs steps (step = 2500). We defined the inversion’s area of influence as the region between the first and last maximum peaks of homozygosity.

To further assess the results obtained with iDlG, we analysed all chromosomes with four additional state-of-the-art methodologies previously described for inversion detection including a) linkage disequilibrium (LD), b) local (i.e., by chromosome) Multi-Dimensional Scaling (local-MDS), c) group genetic differentiation with F_ST_, and d) Tajima’s D analyses. We conducted linkage disequilibrium analysis within each chromosome with the option ‘-r2’ implemented in PLINK v.2.0^100^. We defined windows of 100,000 bp (-ld-window 100,000), and reduced the quantity of data kept to a 10% (-thin 0.1) of all R^2^ values above 0.7 (-ld-window-r2 0.7). This procedure was carried out using a dataset with all samples, and a second dataset including only those individuals previously identified with iDlG to have a homozygous karyotype (both reference and alternative) in the concerned chromosome. Results were visually represented with the R package ‘ggplot2’. We also carried out local-MDS analyses using the ‘cluster’ function of PLINK v.2.0^100^. MDSs plots were generated with the ‘ggplot2’ package. We also selected individuals homokaryotypic for the two arrangements of the inversion in the analysed chromosomes with inversions, as identified with iDlG, and performed an F_ST_ analysis comparing the two groups with VCFTools. We calculated the F_ST_ values using the function ‘-weir-fst-pop’, with a 10,000 bp window size (-fst-window-size 10,000) and a sliding step of 2500 bp (-fst-window-step 2500). Values were graphically represented as a Manhattan plot using the R package ‘CMplot’ v.3.1.3 (Yin, 2017), and we defined the inversion’s influence area according to the region with highest F_ST_ values. Finally, we tested whether these regions identified by iDlG as potentially linked regions were under balancing, disruptive or no selection. To do so, we first calculated global nucleotide diversity (π) and global Tajima’s D using VCFtools, using windows of 10,000 bp (-window-pi 10,000) with a sliding step of 2,500 bp (-window-pi-step 2500) for π, and without sliding step for Tajima’s D (-TajimaD 10,000). Tajima’s D and global nucleotide diversity (π) values were represented as Manhattan plots drawn with ‘CMplot’. We tested if Tajima’s D values were significantly different between linked and non-linked regions, based on the estimated inversion’s influence area identified by iDlG, using a Wilcoxon test with the function ‘wilcox.test’ implemented in R, setting the paired parameter as false (paired = F).

Finally, to validate iDlG’s ability to detect inversions on other species, we used published WGS data from *Cyclopterus lumpus*^29^, as this species has been described to host an inversion in its chromosome 2 of approximately 0.5 Mb^30^. The *C. lumpus* WGS dataset includes 64 specimens from two different locations across the Norwegian coast, with chromosome 2 encompassing 48,608 high-fidelity SNPs. Due to the inversion size and the number of SNPs in chromosome 2, we evaluated the presence of the chromosome 2 inversion using iDlG with windows of 1,000 SNPs and steps of 250 SNPs, since bigger window sizes failed to confidently identify the area of influence of the inversion (data not shown). We also carried out linkage disequilibrium, local-MDS, and F_ST_ approaches as previously described for comparison with iDlG. Results of the four methods were compared to assess consistency among them.

### Population structure and adaptation

To evaluate genomic differentiation among our samples, we conducted MDS analyses based on Identity by State distances using the ‘cluster’ function of PLINK. To test the influence of linked regions in our genome in the population differentiation of our samples, we independently conducted MDS analyses including and excluding the chromosomes hosting linkage groups and plotted the results using the ‘ggplot2’ package.

To detect signals of regional adaptation, F_ST_ values were calculated between all the Atlantic localities except NC (see Results) and all Pacific localities. We searched for outlier F_ST_ values using the function rosnerTest of the R package EnvStats v.2.8.1^101^, which provides a significance value to the outlier values, setting as the number of suspected outliers (parameter k) all those F_ST_ values 1.5 times the interquartile range and a minimum p-value (parameter alpha) of 0.01. Manhattan plots were obtained for the F_ST_ values across the genome with the package ‘CMplot’ and outlier values were highlighted.

### Global mitochondrial genome diversity

We used the filtered WGS-SR data of all individuals, including the individual of the reference genome (BCN-Ref) and the 24 individuals sampled worldwide, to assemble individual mitogenomes with NOVOplasty v.4.2^102^. We used the publicly available *S. plicata* mitogenome NC_013565.1 as reference, a COI sequence as a seed (HQ916426.1), and a k-mer size of k = 33. Mitogenomes were annotated using MITOS2^103^, as implemented in the Galaxy portal^104^, with default parameters and the mitochondrial ascidian genetic code, followed by manual curation with the browser Geneious Prime (https://www.geneious.com/). The mitochondrial genomes of the ascidian species *S. clava* (NC_037072), *Botryllus schlosseri* (NC_021463), and *Ciona robusta* (NC_034372) were downloaded for comparison. All protein-coding genes were extracted from the mitogenomes and independently aligned with MAFFT^105^. To identify phylogenetic relationships between individual mitogenomes, independent gene trees were obtained for each protein-coding and ribosomal gene using maximum likelihood approaches with IQ-TREE2 v.2.2.0^106^, performing 50,000 iterations of ultra-fast bootstrap (-b 50,000), without codon position partitioning, and using an evolutionary model GTR + I + G for all of them. We concatenated all resulting gene trees, and generated a consensus gene tree (supertree) using ASTRAL v.5.5.7^107^. A second approach was conducted by concatenating the same genes in a single matrix (supermatrix), and running IQ-TREE2 using the same parameters as previously described. After phylogenetic reconstruction, three different mitogroups were identified (see Results). Alignment of the whole mitochondrial sequences was performed using the option L-INS-i from MAFFT. MEGA11^108^ was used to calculate the whole mitogenome genetic p-distance among main clades by pairwise comparison with pairwise deletion of indel regions. To identify cyto-nuclear associations, we calculated nuclear F_ST_ values between the two most abundant mitogenomic groups in 10,000 bp windows with 2500 bp sliding steps (see Results). In the analysis we included only individuals of the localities with an equal number of representatives of the two mitogenomic groups to reduce the locality and geography effect. We searched for outlier F_ST_ values using the function rosnerTest of the R package EnvStats, with parameter settings as described above. Manhattan plots were obtained for the F_ST_ values across the genome with the package ‘CMplot’ and outlier values were highlighted.

### Functional analyses of candidate adaptive genome regions

We evaluated the functionality of the genes potentially driving *S. plicata*’s adaptive success considering (1) loci within linkage regions, (2) candidate loci identified in regional adaptation, and (3) loci in nuclear regions identified in cyto-nuclear associations. To do so, we identified the function of genes in the areas of influence of inversions identified by iDlG, LD, and F_ST_. For regional adaptation and cyto-nuclear association we focused on significant chromosomal areas larger than 50 kb. We first used eggNOG-mapper^96^ to functionally characterize our annotated genes by obtaining their Gene Ontology Terms (GO terms). We used default parameters, which consider all types of orthologous relationships to maximize the transfer of functional annotations.

1. Linkage regions: We tested for functional enrichment of genes within linked regions. To evaluate differences between linkage detection techniques, we repeated the following procedures considering the area of influence recovered with iDlG, LD, and F_ST_ for comparison. For each stretch presenting a linkage region, we generated two gene lists: one list contained the gene IDs found inside the considered linked region, whereas the other list included the gene IDs across the whole genome excluding the genes in the previous list. To detect functional enrichment, FatiGO^109^ was used to compare the GO terms in the two gene lists (inside and outside each linked region). Enriched GO terms were functionally categorized with the REVIGO website^110^. A table with the resulting clusters of biological functions was provided. The absolute number of most abundant biological functions was graphically represented with the ‘ggplot2’ package. Finally, the locations of the genes within inversions with enriched GO terms were identified in the F_ST_ Manhattan plot. In order to find functions overall enriched in all linked regions, we compared a dataset including the genes found within the four linkage regions based on iDlG, with the genes in the regions of the genome not showing linkage.
2. Regional adaptation: We focused on all genes within a region in chromosome 3 with a large number of outlier F_ST_ values differentiating Atlantic and Pacific localities (see results). We selected the GO terms associated with the genes included in the chromosomal region and functionally categorized them with REVIGO. We evaluated all the functions potentially involved in regional adaptation. A ‘TreeMap’ of the biological functions was plotted with the R package “treemap v.2.4-4” (https://cran.r-project.org/web/packages/treemap/index.html).
3. Cyto-nuclear association: We selected all the GO terms associated with the genes included in the regions of chromosomes 12 and 14 with large numbers of outlier F_ST_ values in the cyto-nuclear association study (see results) and functionally categorized them with REVIGO. We evaluated all the functions potentially involved in cyto-nuclear associations. In this case, we selected not only the biological function, but also the cellular components of the GO terms to assess if the genes produced in the nucleus could be active in the mitochondria. TreeMaps of both datasets were plotted with the “treemap” R package. Additionally, we extracted the sequence of the genes falling in the highest F_ST_ windows with gffREAD v.0.12.8^111^, since these genes would likely be the main drivers of the differentiation between the groups being compared, and identified the name and type of the specific protein using BLAST’s megablast algorithm against NCBI’s nt database.

## Supplementary Information

Below is the link to the electronic supplementary material.

Supplementary Material 1

Supplementary Material 2

## Data Availability

All raw data and both reference genome and mitochondrial genome assemblies have been uploaded to ENA (BioProjects PRJEB67507 and PRJEB67519). All custom scripts, all data used in this study, and nuclear genome and mitogenome annotations have been made available at GitHub (https://github.com/CGaliaCamps/Splicata_genomes/). The software iDlG, necessary for detection of chromosomal linkage groups, can be independently found at Github (https://github.com/CGaliaCamps/iDlG). All tools and their corresponding versions used for this study are available in the “Material & Methods” section.

## References

[1] Roy, H. E. et al. IPBES invasive alien species assessment: Summary for policymakers. Preprint at 10.5281/ZENODO.7430692 (2023).

[2] IPBES. Summary for policymakers of the global assessment report on biodiversity and ecosystem services. Preprint at 10.5281/ZENODO.3553579 (2019).

[3] Marín-Capuz, G. et al*.* Incipient range expansion of green turtles in the Mediterranean. *Mol. Ecol.* e17790 (2025) 10.1111/mec.17790. 10.1111/mec.17790 PMC12100597 40377080

[4] Luna-Ortiz, A. et al. New colonisers drive the increase of the emerging loggerhead turtle nesting in western Mediterranean. *Sci. Rep.* **14**, 1506 (2024). 38233518 10.1038/s41598-024-51664-w PMC10794258

[5] Hoberg, E. P. & Brooks, D. R. Evolution in action: Climate change, biodiversity dynamics and emerging infectious disease. *Philos. Trans. R. Soc. Lond. B Biol. Sci.* **370** (2015). 10.1098/rstb.2013.0553 PMC4342959 25688014

[6] North, H. L., McGaughran, A. & Jiggins, C. D. Insights into invasive species from whole-genome resequencing. *Mol. Ecol.* **30**, 6289–6308 (2021). 34041794 10.1111/mec.15999

[7] Theissinger, K. et al. How genomics can help biodiversity conservation. *Trends Genet.*10.1016/j.tig.2023.01.005 (2023). 36801111 10.1016/j.tig.2023.01.005

[8] Formenti, G. et al. The era of reference genomes in conservation genomics. *Trends Ecol. Evol.* **37**, 197–202 (2022). 35086739 10.1016/j.tree.2021.11.008 PMC13065249

[9] Corominas, M. et al. The Catalan initiative for the Earth BioGenome Project: contributing local data to global biodiversity genomics. *NAR Genom. Bioinform.* **6**, Iqae075 (2024). 10.1093/nargab/lqae075 PMC11252852 39022326

[10] Valiente-Mullor, C. et al. One is not enough: On the effects of reference genome for the mapping and subsequent analyses of short-reads. *PLoS Comput. Biol.* **17**, e1008678 (2021). 33503026 10.1371/journal.pcbi.1008678 PMC7870062

[11] Eisenstein, M. Every base everywhere all at once: Pangenomics comes of age. *Nature* **616**, 618–620 (2023). 37072518 10.1038/d41586-023-01300-w

[12] Pegueroles, C., Pascual, M. & Carreras, C. Going beyond a reference genome in conservation genomics. *Trends Ecol. Evol.*10.1016/j.tree.2023.11.009 (2023). 38040545 10.1016/j.tree.2023.11.009

[13] Hohenlohe, P. A., Funk, W. C. & Rajora, O. P. Population genomics for wildlife conservation and management. *Mol. Ecol.* **30**, 62–82 (2021). 33145846 10.1111/mec.15720 PMC7894518

[14] Berdan, E. L. et al. How chromosomal inversions reorient the evolutionary process. *J. Evol. Biol.*10.1111/jeb.14242 (2023). 37942504 10.1111/jeb.14242

[15] Mérot, C., Oomen, R. A., Tigano, A. & Wellenreuther, M. A roadmap for understanding the evolutionary significance of structural genomic variation. *Trends Ecol. Evol.* **35**, 561–572 (2020). 32521241 10.1016/j.tree.2020.03.002

[16] Li, H. & Ralph, P. Local PCA shows how the effect of population structure differs along the genome. *Genetics* **211**, 289–304 (2019). 30459280 10.1534/genetics.118.301747 PMC6325702

[17] Lotterhos, K. E. The effect of neutral recombination variation on genome scans for selection. *G3* **9**, 1851–1867 (2019). 30971391 10.1534/g3.119.400088 PMC6553532

[18] Mérot, C. et al. Locally adaptive inversions modulate genetic variation at different geographic scales in a seaweed fly. *Mol. Biol. Evol.* **38**, 3953–3971 (2021). 33963409 10.1093/molbev/msab143 PMC8382925

[19] IPBES. Summary for policymakers of the global assessment report on biodiversity and ecosystem services. Preprint at 10.5281/ZENODO.3553579 (2019).

[20] Roy, H. E. et al. IPBES Invasive Alien Species assessment: Summary for policymakers. Preprint at 10.5281/ZENODO.7430692 (2023).

[21] Hohnen, R. et al. Abundance and detection of feral cats decreases after severe fire on Kangaroo Island, Australia. *Aust. Ecol.*10.1111/aec.13294 (2023).

[22] Touchard, F., Simon, A., Bierne, N. & Viard, F. Urban rendezvous along the seashore: Ports as Darwinian field labs for studying marine evolution in the Anthropocene. *Evol. Appl.* **16**, 560–579 (2023). 36793678 10.1111/eva.13443 PMC9923491

[23] Barros, R. Human-mediated global dispersion of *Styela plicata* (Tunicata, Ascidiacea). *Aquat. Invasions* **4**, 45–57 (2009).

[24] Pineda, M. C., López-Legentil, S. & Turon, X. The whereabouts of an ancient wanderer: Global phylogeography of the solitary ascidian *Styela plicata*. *PLoS ONE* **6**, e25495 (2011). 21966535 10.1371/journal.pone.0025495 PMC3179514

[25] Pineda, M. C., López-Legentil, S. & Turon, X. Year-round reproduction in a seasonal sea: Biological cycle of the introduced ascidian *Styela plicata* in the Western Mediterranean. *Mar. Biol.* **160**, 221–230 (2013).

[26] Casso, M. et al. Seasonal patterns of settlement and growth of introduced and native ascidians in bivalve cultures in the Ebro Delta (NE Iberian Peninsula). *Reg. Stud. Mar. Sci.* **23**, 12–22 (2018).

[27] Taylor, K. M. The chromosomes of some lower chordates. *Chromosoma* **21**, 181–188. 10.1007/bf00343643 (1967). 5603286 10.1007/BF00343643

[28] Wei, J. et al. Genomic basis of environmental adaptation in the leathery sea squirt (*Styela clava*). *Mol. Ecol. Resour.* **20**, 1414–1431 (2020). 32531855 10.1111/1755-0998.13209 PMC7540406

[29] Horaud, M. et al. Allochrony in Atlantic lumpfish: Genomic and otolith shape divergence between spring and autumn spawners. *Ecol. Evol.* **15**, e70946 (2025). 39958818 10.1002/ece3.70946 PMC11826085

[30] Langille, B. L. et al. Trans-Atlantic genomic differentiation and parallel environmental and allelic variation in Lumpfish (*Cyclopterus lumpus*). *ICES J. Mar. Sci.* **81**, 1025–1038 (2024).

[31] Brockhurst, M. A. et al. The ecology and evolution of pangenomes. *Curr. Biol.* **29**, R1094–R1103 (2019). 31639358 10.1016/j.cub.2019.08.012

[32] Galià-Camps, C., Enguídanos, A., Turon, X., Pascual, M. & Carreras, C. The past, the recent, and the ongoing evolutionary processes of the worldwide invasive ascidian *Styela plicata*. *Mol. Ecol.* e17502 (2024) 10.1111/mec.17502. 10.1111/mec.17502 39205460

[33] Pegueroles, C., Ordóñez, V., Mestres, F. & Pascual, M. Recombination and selection in the maintenance of the adaptive value of inversions. *J. Evol. Biol.* **23**, 2709–2717 (2010). 20964762 10.1111/j.1420-9101.2010.02136.x

[34] Jones, F. C. et al. The genomic basis of adaptive evolution in threespine sticklebacks. *Nature* **484**, 55–61 (2012). 22481358 10.1038/nature10944 PMC3322419

[35] Faria, R. et al. Multiple chromosomal rearrangements in a hybrid zone between *Littorina saxatilis* ecotypes. *Mol. Ecol.* **28**, 1375–1393 (2019). 30537056 10.1111/mec.14972 PMC6518922

[36] Tepolt, C. K., Grosholz, E. D., de Rivera, C. E. & Ruiz, G. M. Balanced polymorphism fuels rapid selection in an invasive crab despite high gene flow and low genetic diversity. *Mol. Ecol.* **31**, 55–69 (2022). 34431151 10.1111/mec.16143

[37] Hudson, J. *et al.* Genomics-informed models reveal extensive stretches of coastline under threat by an ecologically dominant invasive species. *Proc. Natl. Acad. Sci. U. S. A.* **118** (2021). 10.1073/pnas.2022169118 PMC8201766 34083434

[38] Casso, M., Turon, X. & Pascual, M. Single zooids, multiple loci: Independent colonisations revealed by population genomics of a global invader. *Biol. Invasions* **21**, 3575–3592 (2019).

[39] Bernheim, A. & Sorek, R. The pan-immune system of bacteria: antiviral defence as a community resource. *Nat. Rev. Microbiol.* **18**, 113–119 (2020). 31695182 10.1038/s41579-019-0278-2

[40] Shlesinger, T. & Loya, Y. Sexual reproduction of scleractinian corals in mesophotic coral ecosystems vs. Shallow reefs. In *Coral Reefs of the World* 653–666 (Springer, Cham, 2019). 10.1007/978-3-319-92735-0_35.

[41] Hu, H. et al. *Amborella *gene presence/absence variation is associated with abiotic stress responses that may contribute to environmental adaptation. *New Phytol.* **233**, 1548–1555 (2022). 34328223 10.1111/nph.17658 PMC9292397

[42] Coffin, J. L., Kelley, J. L., Jeyasingh, P. D. & Tobler, M. Impacts of heavy metal pollution on the ionomes and transcriptomes of Western mosquitofish (*Gambusia affinis*). *Mol. Ecol.* **31**, 1527–1542 (2022). 35000238 10.1111/mec.16342

[43] Zivanovic, G., Arenas, C. & Mestres, F. The adaptive value of chromosomal inversions and climatic change-studies on the natural populations of from the Balkans. *Insects* **14**, (2023). 10.3390/insects14070596 PMC10380441 37504602

[44] Balanyá, J., Oller, J. M., Huey, R. B., Gilchrist, G. W. & Serra, L. Global genetic change tracks global climate warming in *Drosophila subobscura*. *Science* **313**, 1773–1775 (2006). 16946033 10.1126/science.1131002

[45] Rodríguez-Trelles, F., Tarrío, R. & Santos, M. Genome-wide evolutionary response to a heat wave in *Drosophila*. *Biol. Lett.* **9**, 20130228 (2013). 23740296 10.1098/rsbl.2013.0228 PMC3730632

[46] Simões, P., Calabria, G., Picão-Osório, J., Balanyà, J. & Pascual, M. The genetic content of chromosomal inversions across a wide latitudinal gradient. *PLoS ONE* **7**, e51625 (2012). 23272126 10.1371/journal.pone.0051625 PMC3525579

[47] Dalongeville, A., Benestan, L., Mouillot, D., Lobreaux, S. & Manel, S. Combining six genome scan methods to detect candidate genes to salinity in the Mediterranean striped red mullet (*Mullus surmuletus*). *BMC Genomics* **19**, 217 (2018). 29580201 10.1186/s12864-018-4579-z PMC5870821

[48] Carreras, C. et al. The two sides of the Mediterranean: Population genomics of the black sea urchin *Arbacia lixula* (Linnaeus, 1758) in a warming sea. *Front. Mar. Sci.* **8** (2021).

[49] Wang, T. et al. The Human Pangenome Project: A global resource to map genomic diversity. *Nature* **604**, 437–446 (2022). 35444317 10.1038/s41586-022-04601-8 PMC9402379

[50] Vawter, L. & Brown, W. M. Nuclear and mitochondrial DNA comparisons reveal extreme rate variation in the molecular clock. *Science* **234**, 194–196 (1986). 3018931 10.1126/science.3018931

[51] Hill, G. E. Mitonuclear compensatory coevolution. *Trends Genet.* **36**, 403–414 (2020). 32396834 10.1016/j.tig.2020.03.002

[52] Nguyen, T. H. M., Sondhi, S., Ziesel, A., Paliwal, S. & Fiumera, H. L. Mitochondrial-nuclear coadaptation revealed through mtDNA replacements in *Saccharomyces cerevisiae*. *BMC Evol. Biol.* **20**, 128 (2020). 32977769 10.1186/s12862-020-01685-6 PMC7517635

[53] Maszczak-Seneczko, D., Wiktor, M., Skurska, E., Wiertelak, W. & Olczak, M. Delivery of nucleotide sugars to the mammalian Golgi: A very well (un)explained story. *Int. J. Mol. Sci.* **23**, (2022). 10.3390/ijms23158648 PMC9368800 35955785

[54] Burnham-Marusich, A. R. & Berninsone, P. M. Multiple proteins with essential mitochondrial functions have glycosylated isoforms. *Mitochondrion* **12**, 423–427 (2012). 22564751 10.1016/j.mito.2012.04.004 PMC3595188

[55] Scofield, D. G. & Lynch, M. Evolutionary diversification of the Sm family of RNA-associated proteins. *Mol. Biol. Evol.* **25**, 2255–2267 (2008). 18687770 10.1093/molbev/msn175 PMC3888151

[56] Pepling, M. E., Wilhelm, J. E., O’Hara, A. L., Gephardt, G. W. & Spradling, A. C. Mouse oocytes within germ cell cysts and primordial follicles contain a Balbiani body. *Proc. Natl. Acad. Sci. U. S. A.* **104**, 187–192 (2007). 17189423 10.1073/pnas.0609923104 PMC1765432

[57] Jamieson-Lucy, A. & Mullins, M. C. The vertebrate Balbiani body, germ plasm, and oocyte polarity. *Curr. Top. Dev. Biol.* **135**, 1–34 (2019). 31155356 10.1016/bs.ctdb.2019.04.003

[58] Mayjonade, B. et al. Extraction of high-molecular-weight genomic DNA for long-read sequencing of single molecules. *Biotechniques* **61**, 203–205 (2016). 27712583 10.2144/000114460

[59] Ghangal, R., Raghuvanshi, S. & Chand Sharma, P. Isolation of good quality RNA from a medicinal plant seabuckthorn, rich in secondary metabolites. *Plant Physiol. Biochem.* **47**, 1113–1115 (2009). 19804984 10.1016/j.plaphy.2009.09.004

[60] Chueca, L. J. et al. De novo Genome Assembly of the Raccoon Dog (*Nyctereutes procyonoides*). *Front. Genet.* **12**, 658256 (2021). 33995489 10.3389/fgene.2021.658256 PMC8117329

[61] Galbraith, D. W. et al. Rapid flow cytometric analysis of the cell cycle in intact plant tissues. *Science* **220**, 1049–1051 (1983). 17754551 10.1126/science.220.4601.1049

[62] Otto, F. DAPI staining of fixed cells for high-resolution flow cytometry of nuclear DNA. *Methods Cell Biol.* **33**, 105–110 (1990). 1707478 10.1016/s0091-679x(08)60516-6

[63] Danecek, P. *et al.* Twelve years of SAMtools and BCFtools. *Gigascience* **10** (2021). 10.1093/gigascience/giab008 PMC7931819 33590861

[64] De Coster, W., D’Hert, S., Schultz, D. T., Cruts, M. & Van Broeckhoven, C. NanoPack: Visualizing and processing long-read sequencing data. *Bioinformatics* **34**, 2666–2669 (2018). 29547981 10.1093/bioinformatics/bty149 PMC6061794

[65] Bolger, A. M., Lohse, M. & Usadel, B. Trimmomatic: A flexible trimmer for Illumina sequence data. *Bioinformatics* **30**, 2114–2120. 10.1093/bioinformatics/btu170 (2014). 24695404 10.1093/bioinformatics/btu170 PMC4103590

[66] Andrews, S. FastQC: a quality control tool for high throughput sequence data. Preprint at https://github.com/s-andrews/FastQC (2010).

[67] Kolmogorov, M., Yuan, J., Lin, Y. & Pevzner, P. A. Assembly of long, error-prone reads using repeat graphs. *Nat. Biotechnol.* **37**, 540–546 (2019). 30936562 10.1038/s41587-019-0072-8

[68] Walker, B. J. et al. Pilon: an integrated tool for comprehensive microbial variant detection and genome assembly improvement. *PLoS ONE* **9**, e112963 (2014). 25409509 10.1371/journal.pone.0112963 PMC4237348

[69] Guan, D. et al. Identifying and removing haplotypic duplication in primary genome assemblies. *Bioinformatics* **36**, 2896–2898 (2020). 31971576 10.1093/bioinformatics/btaa025 PMC7203741

[70] Durand, N. C. et al. Juicer provides a one-click system for analyzing loop-resolution Hi-C experiments. *Cell Syst.* **3**, 95–98 (2016). 27467249 10.1016/j.cels.2016.07.002 PMC5846465

[71] Dudchenko, O. et al. De novo assembly of the genome using Hi-C yields chromosome-length scaffolds. *Science* **356**, 92–95 (2017). 28336562 10.1126/science.aal3327 PMC5635820

[72] Durand, N. C. et al. Juicebox provides a visualization system for Hi-C contact maps with unlimited zoom. *Cell Syst.* **3**, 99–101 (2016). 27467250 10.1016/j.cels.2015.07.012 PMC5596920

[73] Li, H. Minimap2: Pairwise alignment for nucleotide sequences. *Bioinformatics* **34**, 3094–3100 (2018). 29750242 10.1093/bioinformatics/bty191 PMC6137996

[74] Li, H. & Durbin, R. Fast and accurate short read alignment with Burrows–Wheeler transform. *Bioinformatics* **25**, 1754–1760 (2009). 19451168 10.1093/bioinformatics/btp324 PMC2705234

[75] Okonechnikov, K., Conesa, A. & García-Alcalde, F. Qualimap 2: Advanced multi-sample quality control for high-throughput sequencing data. *Bioinformatics* **32**, 292–294 (2016). 26428292 10.1093/bioinformatics/btv566 PMC4708105

[76] Ewels, P., Magnusson, M., Lundin, S. & Käller, M. MultiQC: Summarize analysis results for multiple tools and samples in a single report. *Bioinformatics* **32**, 3047–3048. 10.1093/bioinformatics/btw354 (2016). 27312411 10.1093/bioinformatics/btw354 PMC5039924

[77] Schell, T. et al. An annotated draft genome for *Radix auricularia* (Gastropoda, Mollusca). *Genome Biol. Evol.* **9**, 585–592 (2017). 10.1093/gbe/evx032 PMC5381561 28204581

[78] Rhie, A., Walenz, B. P., Koren, S. & Phillippy, A. M. Merqury: Reference-free quality, completeness, and phasing assessment for genome assemblies. *Genome Biol.* **21**, 245 (2020). 32928274 10.1186/s13059-020-02134-9 PMC7488777

[79] Dumontier, M. & Hogue, C. W. V. NBLAST: A cluster variant of BLAST for NxN comparisons. *BMC Bioinformatics* **3**, 13 (2002). 12019022 10.1186/1471-2105-3-13 PMC113272

[80] Laetsch, D. R. & Blaxter, M. L. BlobTools: Interrogation of genome assemblies. *F1000Res.* **6** (2017).

[81] Jurka, J. et al. Repbase update, a database of eukaryotic repetitive elements. *Cytogenet. Genome Res.* **110**, 462–467 (2005). 16093699 10.1159/000084979

[82] Flynn, J. M. et al. RepeatModeler2 for automated genomic discovery of transposable element families. *Proc. Natl. Acad. Sci. U. S. A.* **117**, 9451–9457 (2020). 32300014 10.1073/pnas.1921046117 PMC7196820

[83] Smit, A. F. A., Hubley, R. & Green, P. RepeatMasker Open-4.0. 0–8 (2021).

[84] UniProt Consortium. UniProt: A worldwide hub of protein knowledge. *Nucleic Acids Res.* **47**, D506–D515 (2019). 30395287 10.1093/nar/gky1049 PMC6323992

[85] Grabherr, M. G. et al. Full-length transcriptome assembly from RNA-Seq data without a reference genome. *Nat. Biotechnol.* **29**, 644–652. 10.1038/nbt.1883 (2011). 21572440 10.1038/nbt.1883 PMC3571712

[86] Fu, L., Niu, B., Zhu, Z., Wu, S. & Li, W. CD-HIT: Accelerated for clustering the next-generation sequencing data. *Bioinformatics* **28**, 3150–3152 (2012). 23060610 10.1093/bioinformatics/bts565 PMC3516142

[87] Holt, C. & Yandell, M. MAKER2: An annotation pipeline and genome-database management tool for second-generation genome projects. *BMC Bioinform.* **12**, 491 (2011). 10.1186/1471-2105-12-491 PMC3280279 22192575

[88] Stanke, M. et al. AUGUSTUS: Ab initio prediction of alternative transcripts. *Nucleic Acids Res.* **34**, W435–W439 (2006). 16845043 10.1093/nar/gkl200 PMC1538822

[89] Brůna, T., Lomsadze, A. & Borodovsky, M. GeneMark-EP+: eukaryotic gene prediction with self-training in the space of genes and proteins. *NAR Genom Bioinform* **2**, lqaa026 (2020). 10.1093/nargab/lqaa026 PMC7222226 32440658

[90] Korf, I. Gene finding in novel genomes. *BMC Bioinform.* **5**, 59 (2004). 10.1186/1471-2105-5-59 PMC421630 15144565

[91] Chan, P. P., Lin, B. Y., Mak, A. J. & Lowe, T. M. tRNAscan-SE 2.0: improved detection and functional classification of transfer RNA genes. *Nucleic Acids Res.* **49**, 9077–9096 (2021). 34417604 10.1093/nar/gkab688 PMC8450103

[92] Lowe, T. M. & Eddy, S. R. A computational screen for methylation guide snoRNAs in yeast. *Science* **283**, 1168–1171 (1999). 10024243 10.1126/science.283.5405.1168

[93] Wucher, V. et al. FEELnc: a tool for long non-coding RNA annotation and its application to the dog transcriptome. *Nucleic Acids Res.* **45**, e57 (2017). 28053114 10.1093/nar/gkw1306 PMC5416892

[94] Kang, Y.-J. et al. CPC2: a fast and accurate coding potential calculator based on sequence intrinsic features. *Nucleic Acids Res.* **45**, W12–W16 (2017). 28521017 10.1093/nar/gkx428 PMC5793834

[95] Pegueroles, C. et al. Transcriptomic analyses reveal groups of co-expressed, syntenic lncRNAs in four species of the genus *Caenorhabditis*. *RNA Biol.* **16**, 320–329 (2019). 30691342 10.1080/15476286.2019.1572438 PMC6380332

[96] Cantalapiedra, C. P., Hernández-Plaza, A., Letunic, I., Bork, P. & Huerta-Cepas, J. eggNOG-mapper v2: Functional Annotation, Orthology Assignments, and Domain Prediction at the Metagenomic Scale. *Mol. Biol. Evol.* **38**, 5825–5829 (2021). 34597405 10.1093/molbev/msab293 PMC8662613

[97] Danecek, P. et al. The variant call format and VCFtools. *Bioinformatics* **27**, 2156–2158 (2011). 21653522 10.1093/bioinformatics/btr330 PMC3137218

[98] Nachman, M. W. Variation in recombination rate across the genome: evidence and implications. *Curr. Opin. Genet. Dev.* **12**, 657–663 (2002). 12433578 10.1016/s0959-437x(02)00358-1

[99] Wickham, H. ggplot2. *Wiley Interdiscip. Rev. Comput. Stat.* **3**, 180–185. 10.1002/wics.147 (2011).

[100] Purcell, S. et al. PLINK: A tool set for whole-genome association and population-based linkage analyses. *Am. J. Hum. Genet.* **81**, 559–575 (2007). 17701901 10.1086/519795 PMC1950838

[101] Millard, S. P. *EnvStats: An R Package for Environmental Statistics* (Springer, New York, 2013). 10.1007/978-1-4614-8456-1.

[102] Dierckxsens, N., Mardulyn, P. & Smits, G. NOVOPlasty: De novo assembly of organelle genomes from whole genome data. *Nucleic Acids Res.* **45**, e18 (2017). 28204566 10.1093/nar/gkw955 PMC5389512

[103] Bernt, M. et al. MITOS: Improved de novo metazoan mitochondrial genome annotation. *Mol. Phylogenet. Evol.* **69**, 313–319 (2013). 22982435 10.1016/j.ympev.2012.08.023

[104] Jalili, V. et al. The Galaxy platform for accessible, reproducible and collaborative biomedical analyses: 2020 update. *Nucleic Acids Res.* **48**, W395–W402 (2020). 32479607 10.1093/nar/gkaa434 PMC7319590

[105] Katoh, K. & Standley, D. M. MAFFT multiple sequence alignment software version 7: Improvements in performance and usability. *Mol. Biol. Evol.* **30**, 772–780 (2013). 23329690 10.1093/molbev/mst010 PMC3603318

[106] Nguyen, L.-T., Schmidt, H. A., von Haeseler, A. & Minh, B. Q. IQ-TREE: A fast and effective stochastic algorithm for estimating maximum-likelihood phylogenies. *Mol. Biol. Evol.* **32**, 268–274 (2015). 25371430 10.1093/molbev/msu300 PMC4271533

[107] Zhang, C., Rabiee, M., Sayyari, E. & Mirarab, S. ASTRAL-III: Polynomial time species tree reconstruction from partially resolved gene trees. *BMC Bioinform.* **19**, 153 (2018). 10.1186/s12859-018-2129-y PMC5998893 29745866

[108] Tamura, K., Stecher, G. & Kumar, S. MEGA11: Molecular evolutionary genetics analysis version 11. *Mol. Biol. Evol.* **38**, 3022–3027 (2021). 33892491 10.1093/molbev/msab120 PMC8233496

[109] Al-Shahrour, F., Díaz-Uriarte, R. & Dopazo, J. FatiGO: A web tool for finding significant associations of gene ontology terms with groups of genes. *Bioinformatics* **20**, 578–580 (2004). 14990455 10.1093/bioinformatics/btg455

[110] Supek, F., Bošnjak, M., Škunca, N. & Šmuc, T. REVIGO summarizes and visualizes long lists of gene ontology terms. *PLoS ONE* **6**, e21800 (2011). 21789182 10.1371/journal.pone.0021800 PMC3138752

[111] Pertea, G. & Pertea, M. GFF utilities: GffRead and GffCompare. *F1000Res* **9**, 304 (2020). 10.12688/f1000research.23297.1 PMC7222033 32489650

