# Supplementary material for: De novo genome assembly, inversion detection, and worldwide adaptation on the invasive species Styela plicata
Source: Sci Rep. 2025 Nov 18;15:40328. doi: 10.1038/s41598-025-24574-8 (PMC12627437; doi:10.1038/s41598-025-24574-8)
Supplement: Supplementary file 1 — Supplementary Material 1 [file 41598_2025_24574_MOESM1_ESM.pdf]

De novo genome assembly, inversion detection, and worldwide adaptation on the invasive species *Styela plicata*.

Carles Galià-Camps [1,2,*], Tilman Schell [3,4], Cinta Pegueroles [1,2,5,6], Damian Baranski [3,4], Alex Ben Hamadou [3,4], Mathilde Horaud [7], Adrià Antich [8], Xavier Turon [8,+], Marta Pascual [1,2,+], Carola Greve [3,4,+], Carlos Carreras [1,2,+].

1. *Departament de Genètica, Microbiologia i Estadística, Universitat de Barcelona, Avinguda Diagonal 643, 08028 Barcelona, Spain.*
2. Institut de Recerca de la Biodiversitat (IRBio), Universitat de Barcelona (UB)
3. *LOEWE Centre for Translational Biodiversity Genomics (LOEWE-TBG), Senckenberganlage 25, 60325 Frankfurt am Main, Germany.*
4. *Senckenberg Forschungsinstitut und Naturmuseum, Senckenberganlage 25, 60325 Frankfurt am Main, Germany.*
5. *Department of Genetics and Microbiology, Universitat Autònoma de Barcelona, Bellaterra, Barcelona 08193, Spain.*
6. *Institute of Biotechnology and Biomedicine, Universitat Autònoma de Barcelona, Bellaterra, Barcelona 08193, Spain.*
7. *Faculty of Biosciences, Fisheries and Economics, The Norwegian College of Fishery Science, UiT The Arctic University of Norway, Tromsø, Norway.*
87. *Centre d'Estudis Avançats de Blanes (CEAB-CSIC), Accés Cala St. Francesc 14, 17300 Blanes, Girona, Spain.*

*: Corresponding author. cgaliacamps@gmail.com
+: These authors jointly supervised this work

SUPPLEMENTARY FIGURES AND TABLES

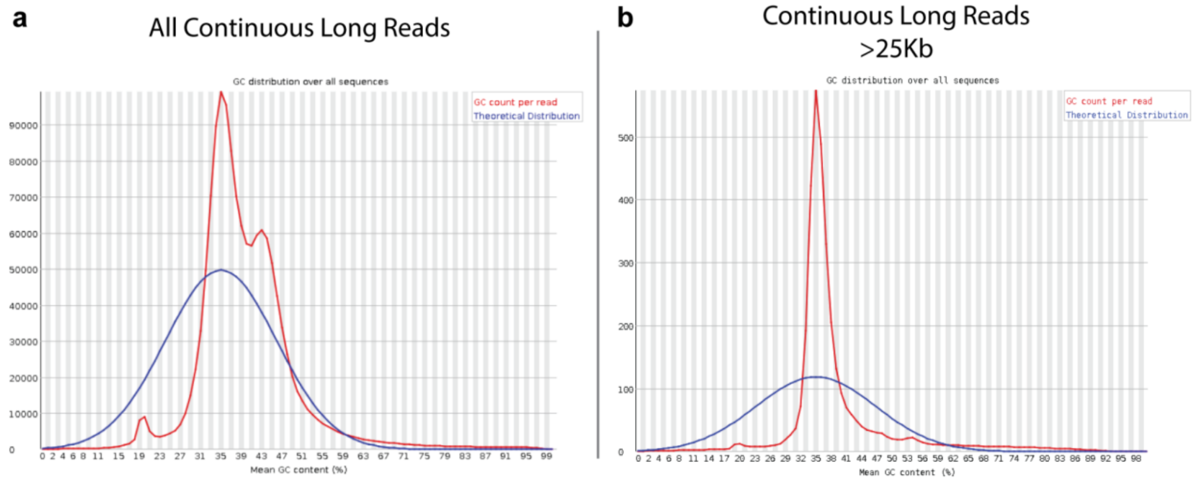

Supplementary Figure 1: PacBio Continuous Long Reads (CLR) GC profiles. a: Sequence data after applying standard quality filters. **b:** Sequence data after applying an additional length filtering (>25Kb). Note that after the length filtering, GC distribution is more uniform, suggesting the removal of possible contamination and/or mitochondrial DNA.

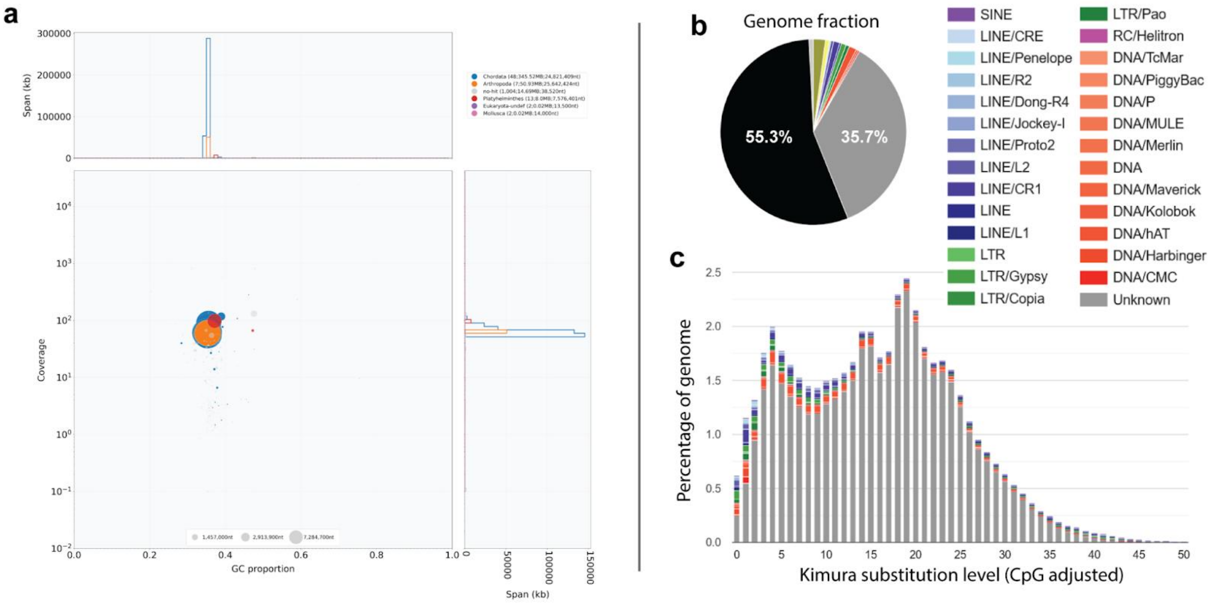

Supplementary Figure 2: Genome assembly features. a: Blob plot of genome assembly scaffolds' size, GC content, coverage, and associated taxonomic group. Circle size and color correspond to sequence length, and taxonomic assignment respectively (Blue=chordata, orange=arthropoda, red=platyhelmintes). Note that all scaffolds (larger circles) are similar in GC content (x axis) and coverage (y axis). **b:** Fraction of the genome annotated as non-repetitive genomic elements (black), unknown Transposable Elements (TE, gray) or identified as different TE families (colored bars). **c:** Frequency distribution of the genetic differences between TEs within families.

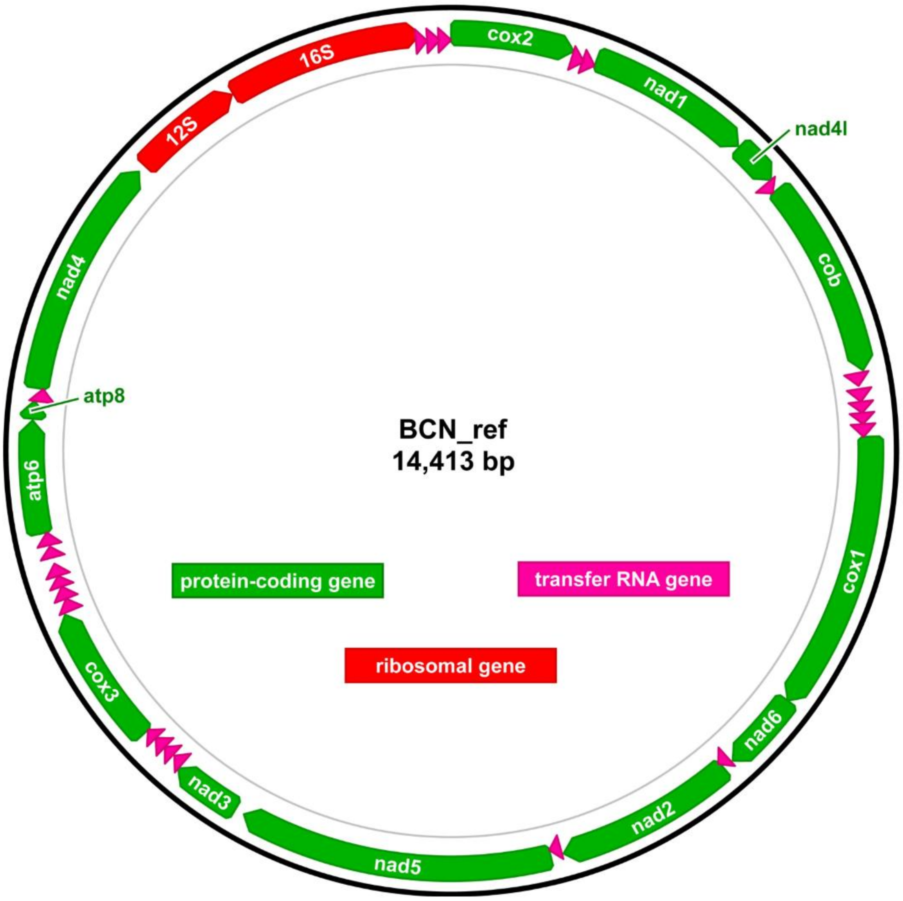

Supplementary Figure 3: Reference genome mitogenome structure.

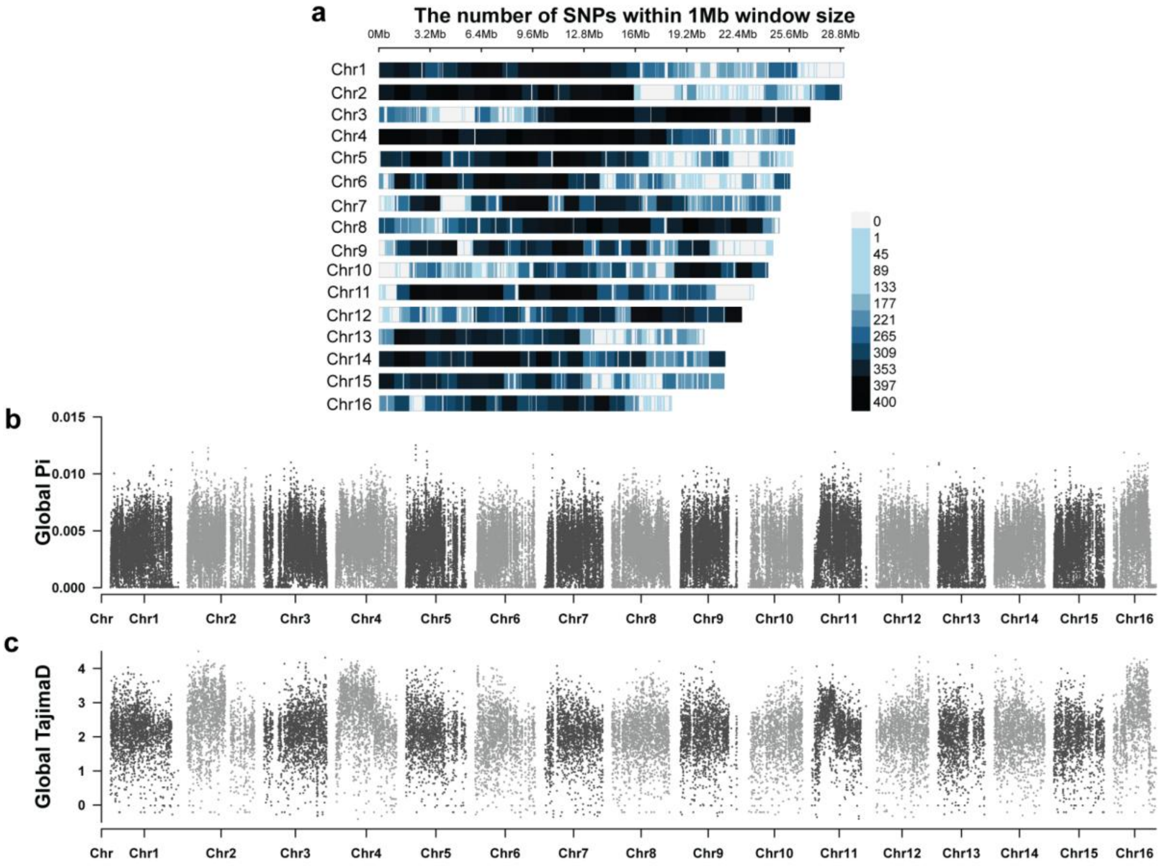

Supplementary Figure 4: Global genetic diversity of the 24 individuals sampled worldwide. a: Filtered SNPs density along the chromosomes. **b: Global diversity (pi)** values along the chromosomes in 10,000 bp windows, with 2500 bp sliding windows. **c: TajimaD** values along the chromosomes in 10,000 bp windows. Note that no outliers were identified for pi and Tajima's D.

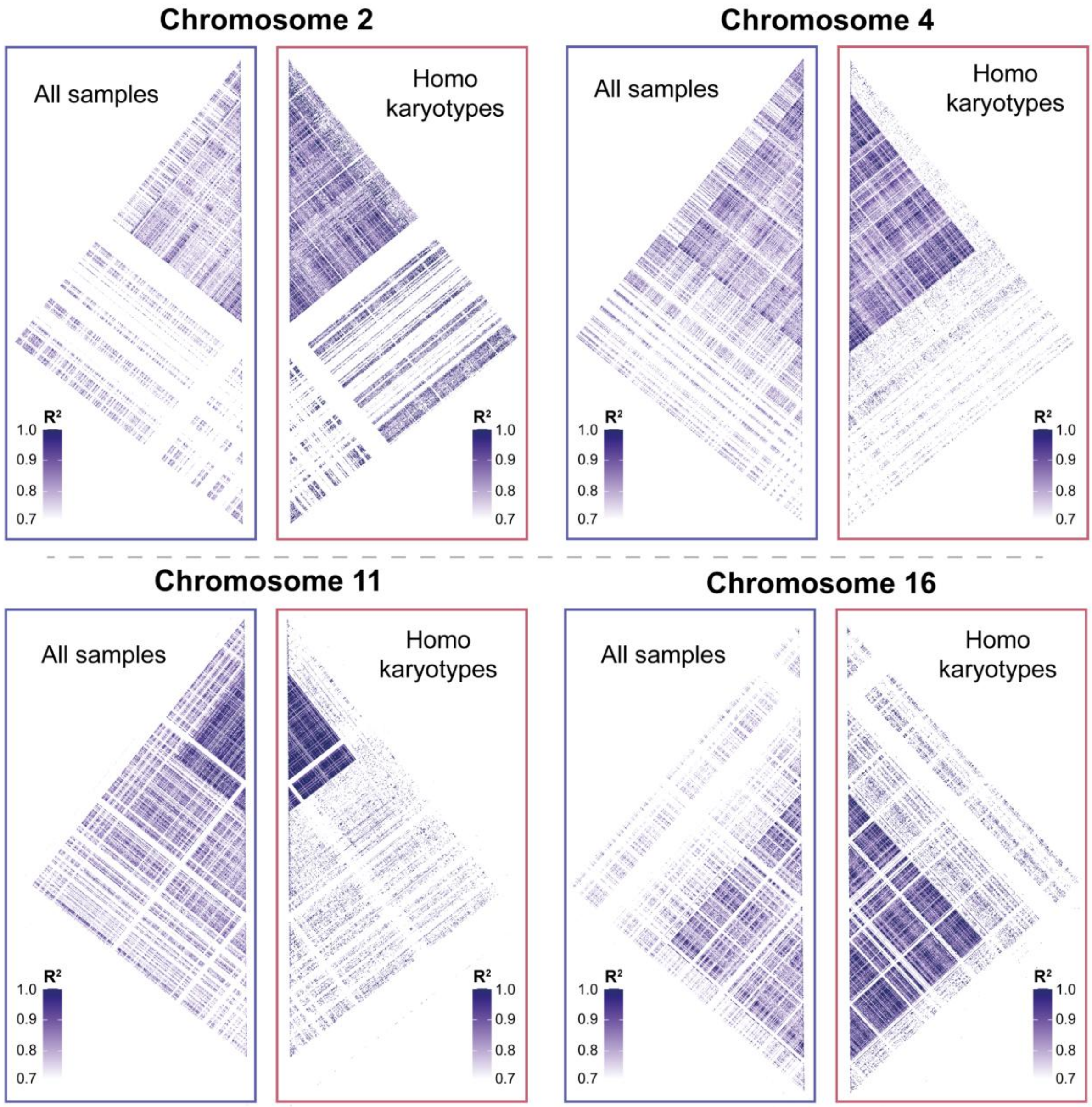

Supplementary Figure 5: Linkage disequilibrium among SNPs with an R^2 above 0.7 summarized by windows of 100 Kb across the four chromosomes hosting a linked region according to iDIG. The left side of each chromosome, inside a blue frame, has been obtained using all samples, whereas the right side of each chromosome, inside a red frame, has been obtained using only homokaryotypes individuals for that specific linked region as detected with iDIG.

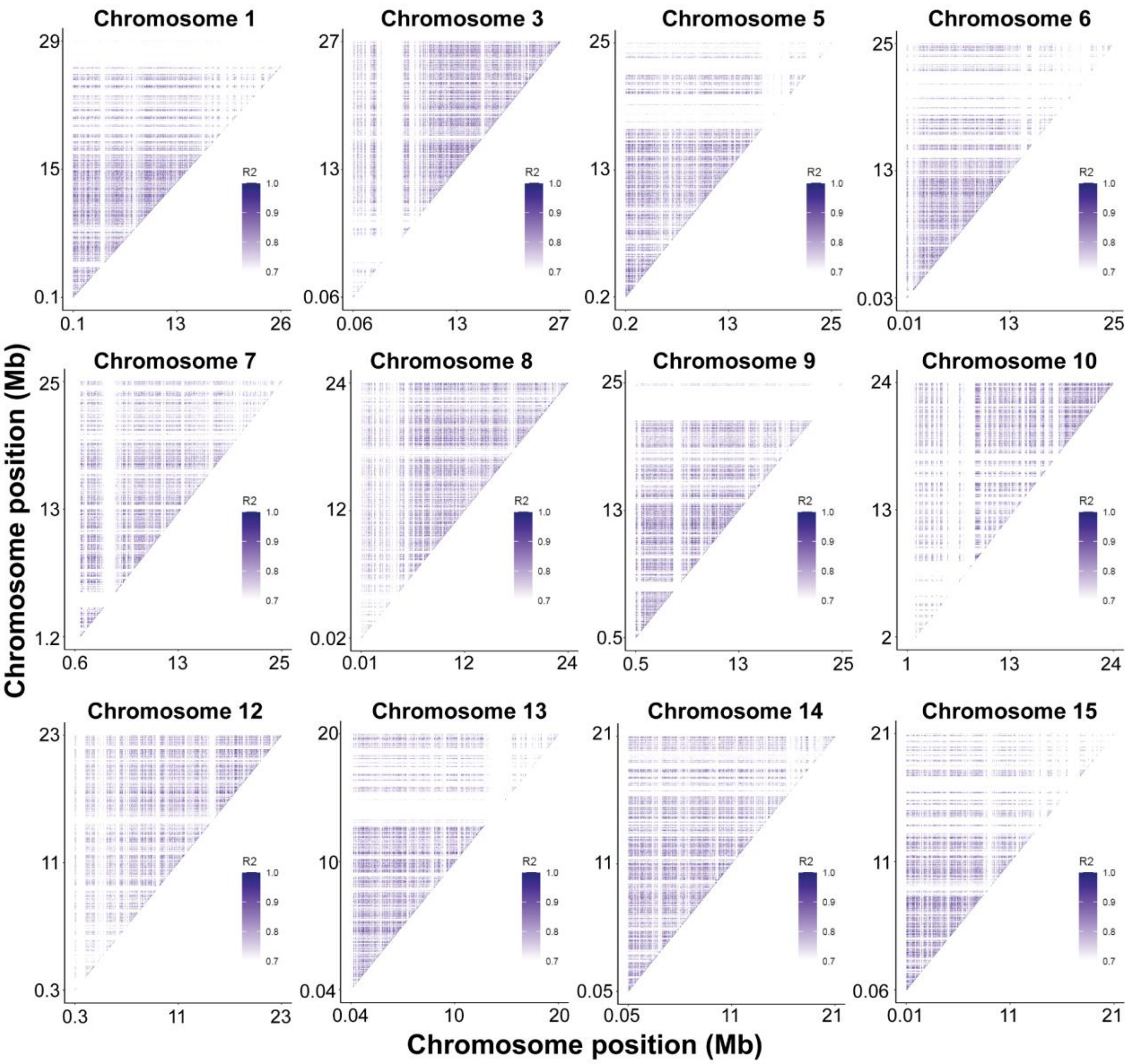

Supplementary Figure 6: Linkage disequilibrium among SNPs with an R^2 above 0.7 summarized by windows of 100 Kb across the chromosomes without inversions according to iDIG. Linkage values have been obtained using all samples.

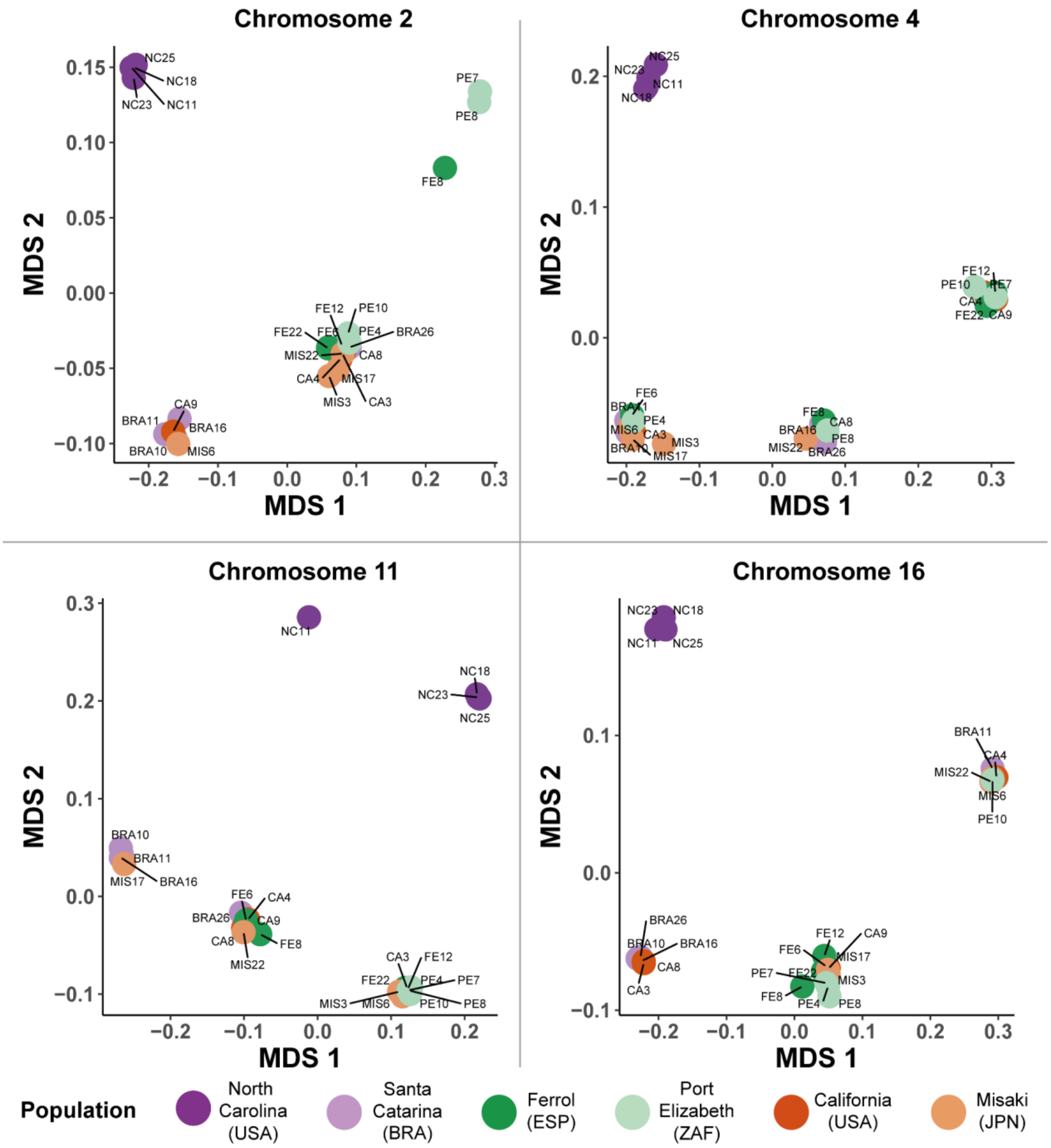

Supplementary Figure 7: Genetic differentiation analysis of the 24 individuals sampled worldwide based only on the genotyping of chromosomes with inversions. MDS analysis plot of all individuals using SNPs in chromosomes with inversions identified with iDIG. Note that each chromosomal karyotype is recovered along the MDS1 (heterokaryotype in the center; homokaryotypes for the two arrangements in the two extremes), whereas the MDS2 reflects differentiation driven by sequence divergence of NC.

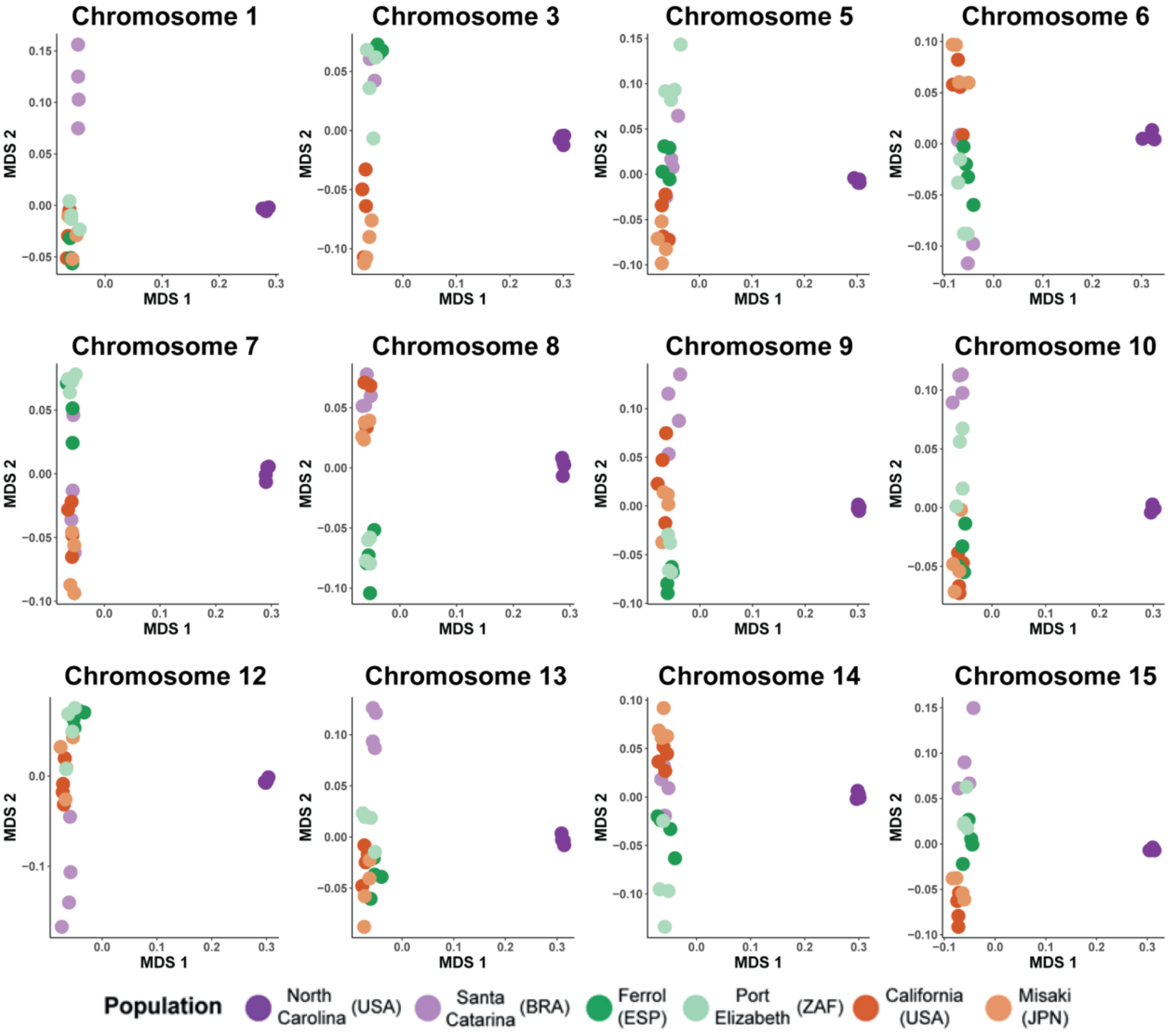

Supplementary Figure 8: Genetic differentiation analysis of the 24 individuals sampled worldwide based only on the genotyping of chromosomes without inversions. Note that the MDS1 reflects sequence differentiation driven by the high divergence of NC, whereas MDS2 reflects population structuring among Atlantic and Pacific Oceans.

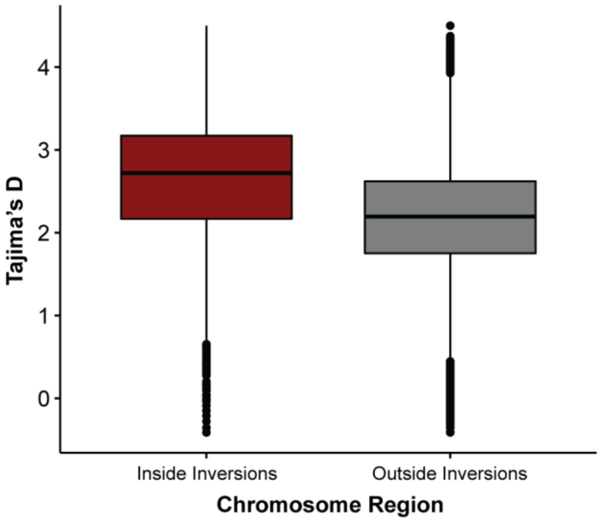

Supplementary Figure 9: Tajima's D values of the 24 individuals sampled worldwide inside and outside the block regions identified as potential inversions based on iDIG (Supplementary Table 6). Box plots' center lines identify the mean value, box limits correspond to the 1st and 3rd quartiles, whiskers are 1.5 interquartile range, and circles outside the whisker range represent outlier values. Tajima's D values differ significantly between the two groups according to Wilcoxon test (W=100,322,610, p-value $< 2.2^{-16}$).

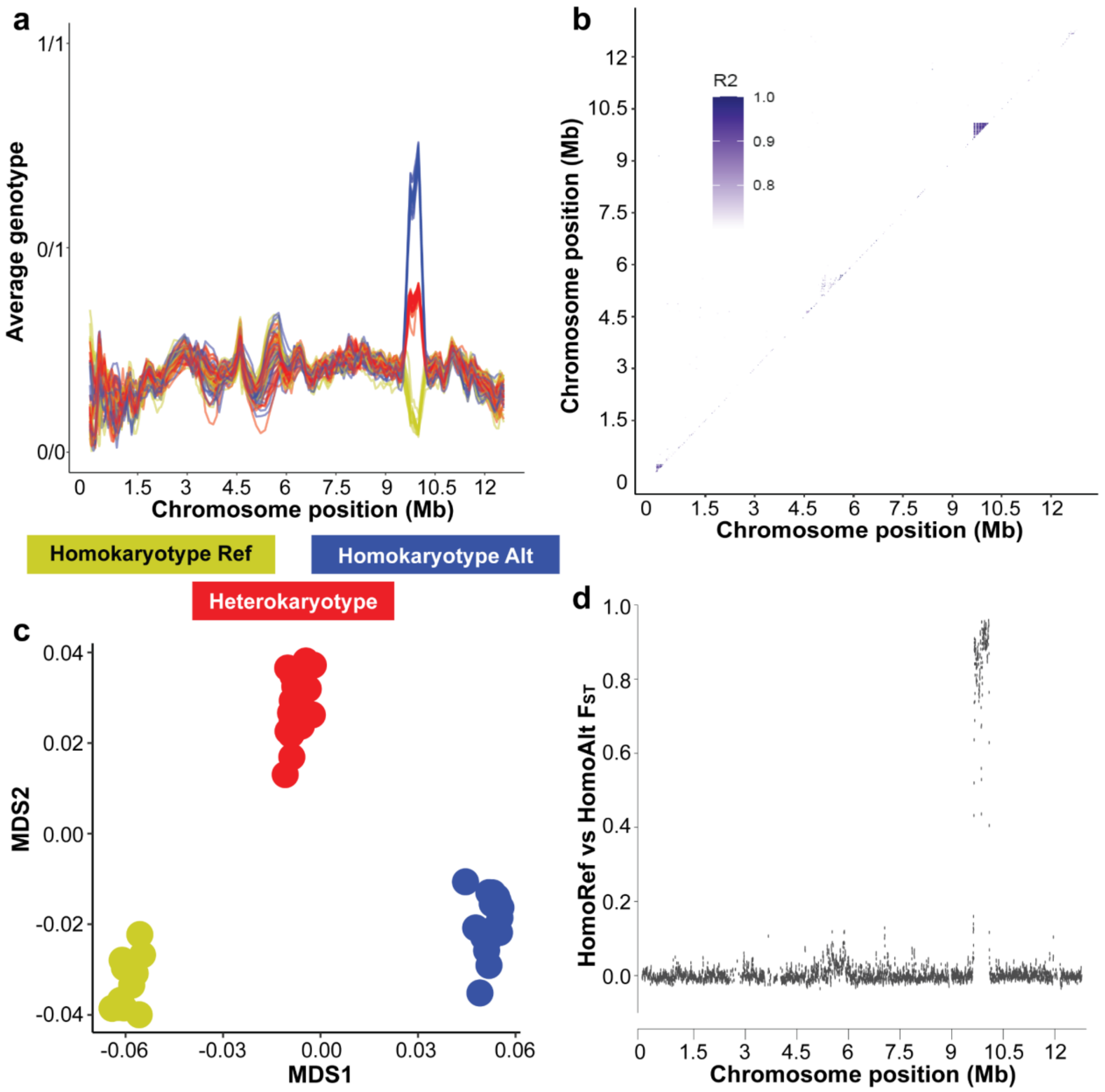

Supplementary Figure 10: Validation of iDIG using a WGS dataset of Cyclopterus lumpus with 64 specimens hosting an inversion on Chromosome 2. a: Average genotype for each individual across all chromosomes based on 1,000 SNPs windows with 250 SNPs sliding window inferred with iDIG. **b:** Linkage disequilibrium among SNPs with an R^2 above 0.7 summarized by windows of 100 Kb. **c:** MDS analysis plot of the 64 individuals of *C.lumpus*. **d:** FST values between individuals from homokaryotypes for the reference allele and homokaryotypes for the alternative allele in 10,000 bp windows, with 2500 bp sliding windows.

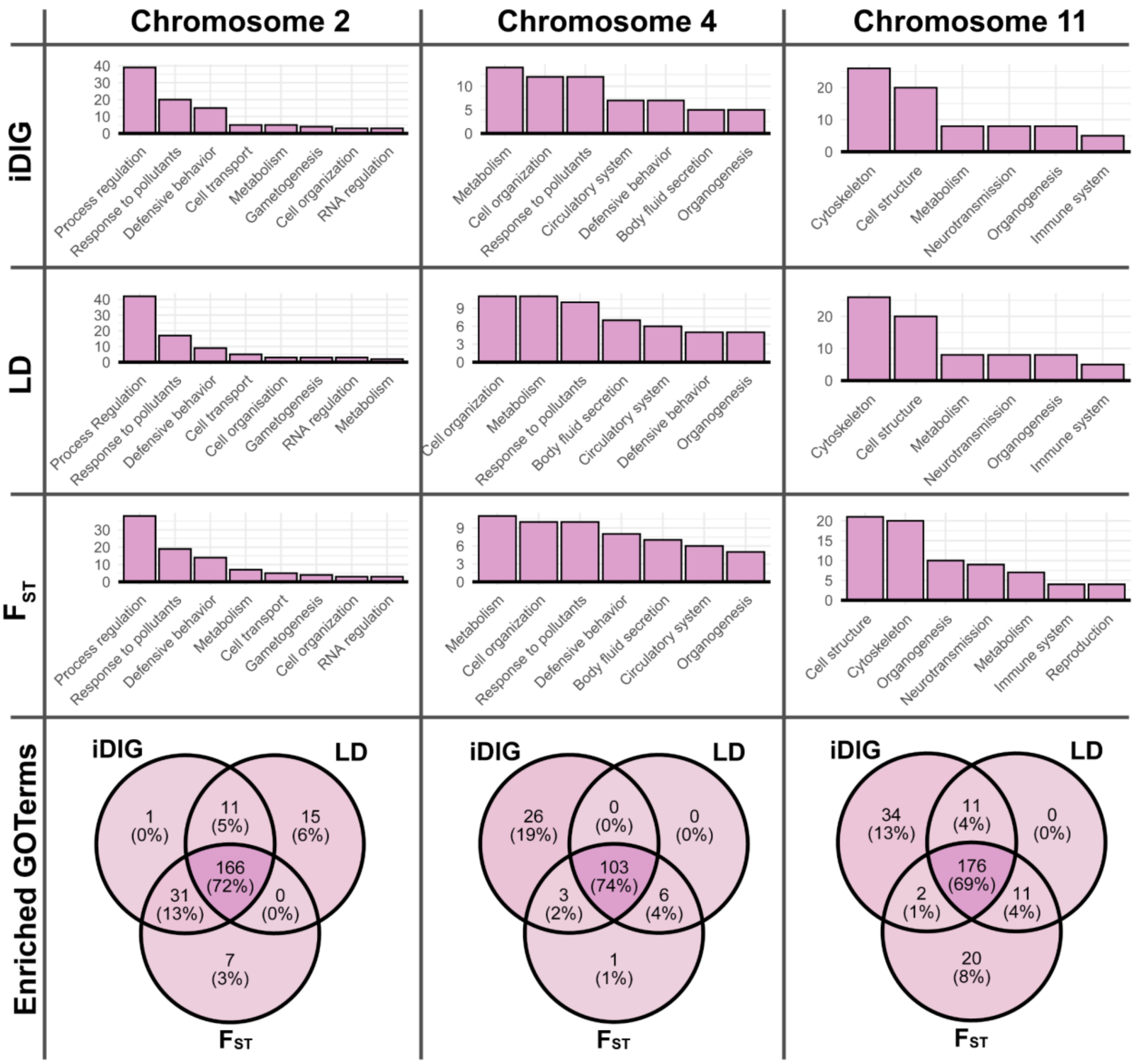

Supplementary Figure 11: Comparison of enriched GOTerms for genes identified in the area of influence of inversions as detected with iDIG, linkage disequilibrium (LD) and F_{ST} methods. The x-axis displays the major functional clusters being enriched within each linked region, and the y-axis displays the number of enriched GO terms belonging to each major functional cluster. Venn diagrams indicate the number of genes with enriched functions being shared among the different methodologies. Note that no enrichment was detected for chromosome 16.

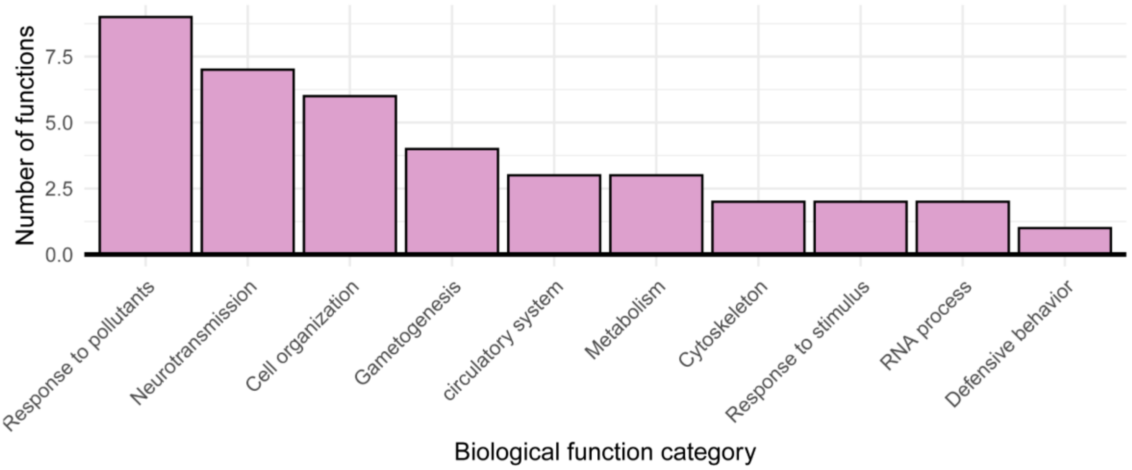

Supplementary Figure 12: Enriched functions considering together all four inversions identified by iDIG. The x-axis displays the major functional clusters being enriched, and the y-axis displays the number of enriched GO terms belonging to each major functional cluster.

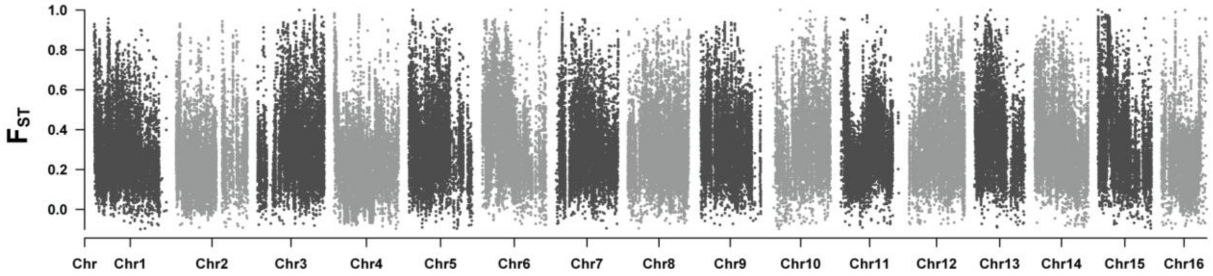

Supplementary Figure 13: Manhattan plot depicting F_{ST} values between individuals from North Carolina and individuals of all remaining populations in 10,000 bp windows, with 2500 bp sliding windows.

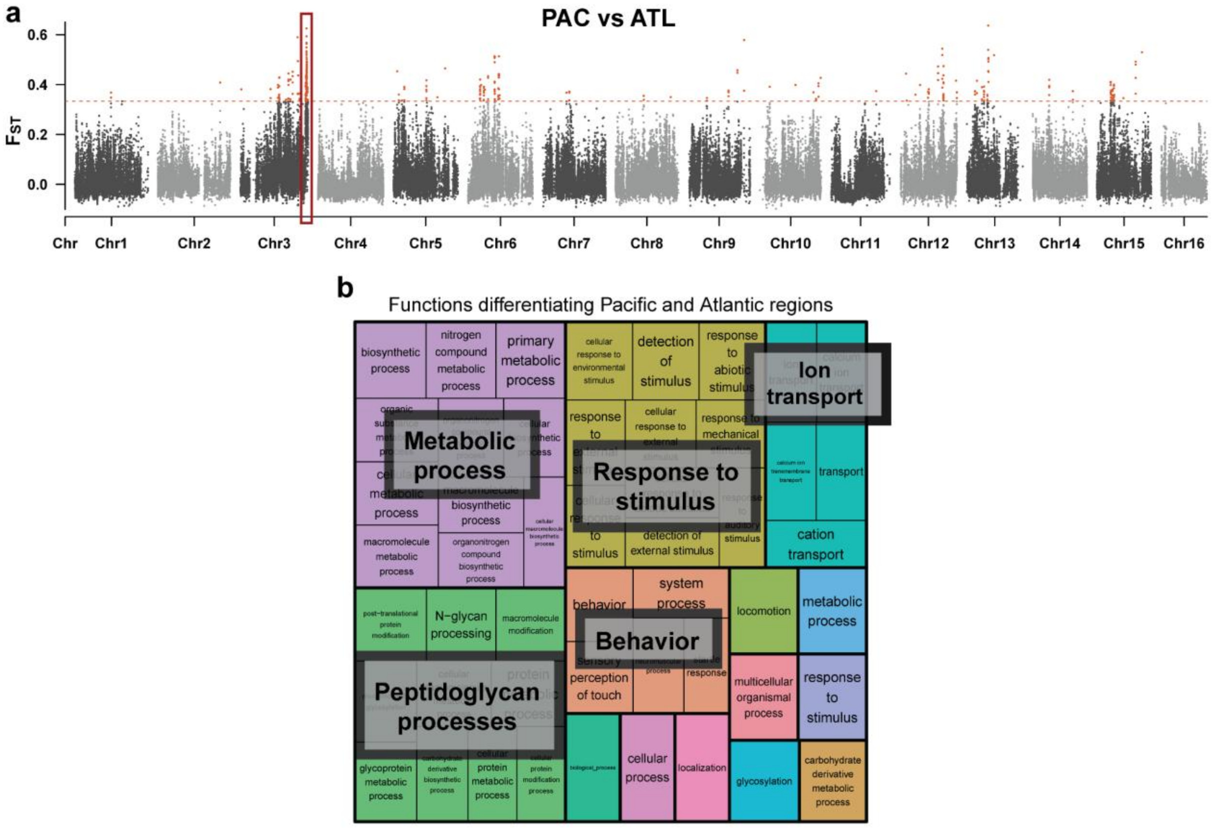

Supplementary Figure 14: Signals of regional adaptation between Atlantic and Pacific individuals. a: F_{ST} values between both groups in 10,000 bp windows, with 2500 bp sliding windows**.** Orange dots represent significant outlier F_{ST} values. The red rectangle indicates the region used for functional analyses. **b:** Biological functions inside the region driving Atlanto-Pacific divergence in chromosome 3. Note that North Carolina individuals were excluded from the analysis since their genetic divergence masks genetic differentiation among the remaining individuals.

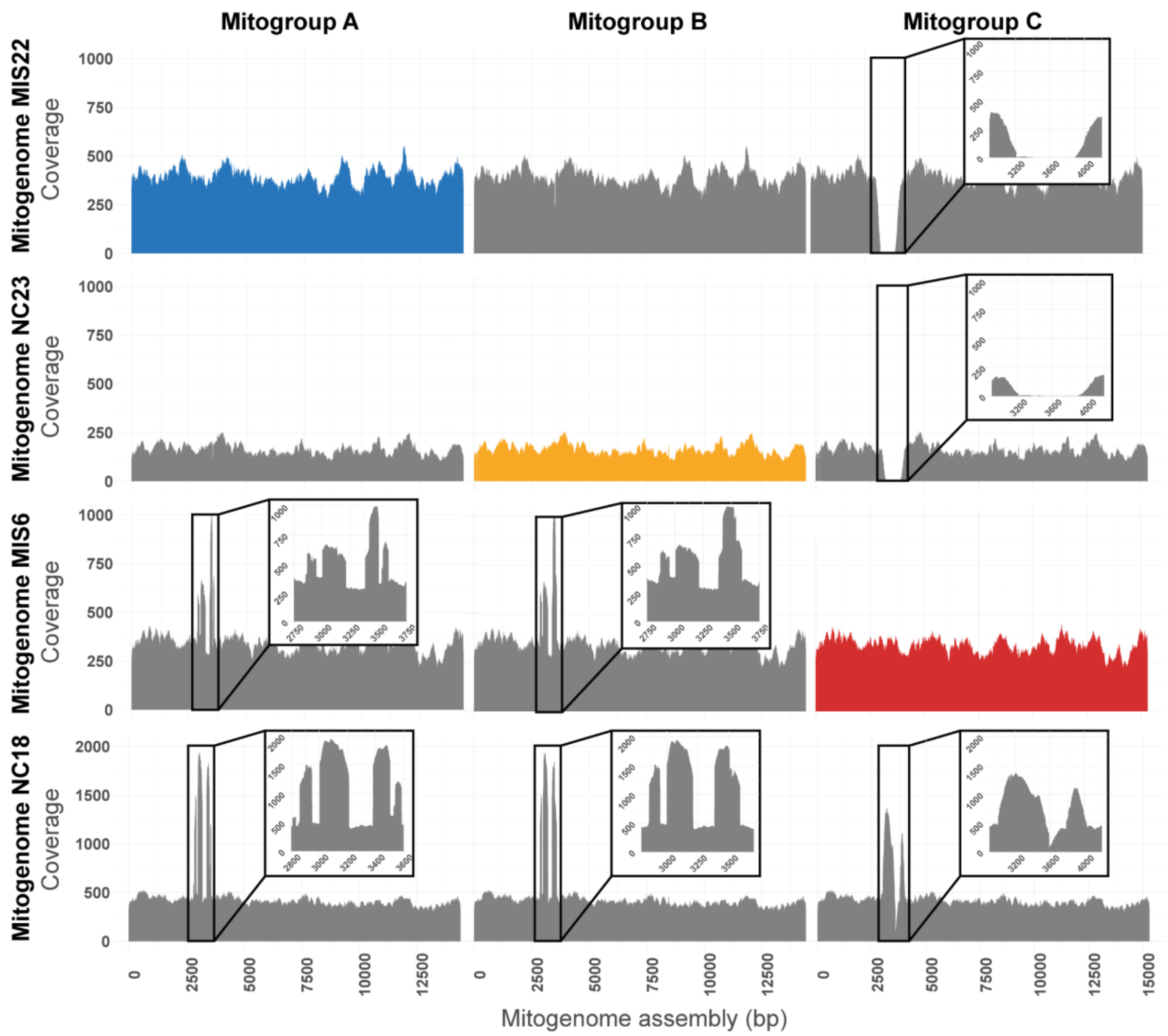

Supplementary Figure 15: Mitogenome mapping profiles. Each column corresponds to an individual representative of each main mitogroup sequence, and rows identify the sample's reads used to generate mapping profiles. Individual MIS22, NC23, and MIS6 were randomly selected as representatives for the mitogroup A, B, and C respectively. Regions with non-uniform coverage were plotted separately for visualization accuracy. NC18 was the only individual whose mitogenome could not be circularized and its reads have been mapped to each of the other three mitogenomes for coverage visualization.

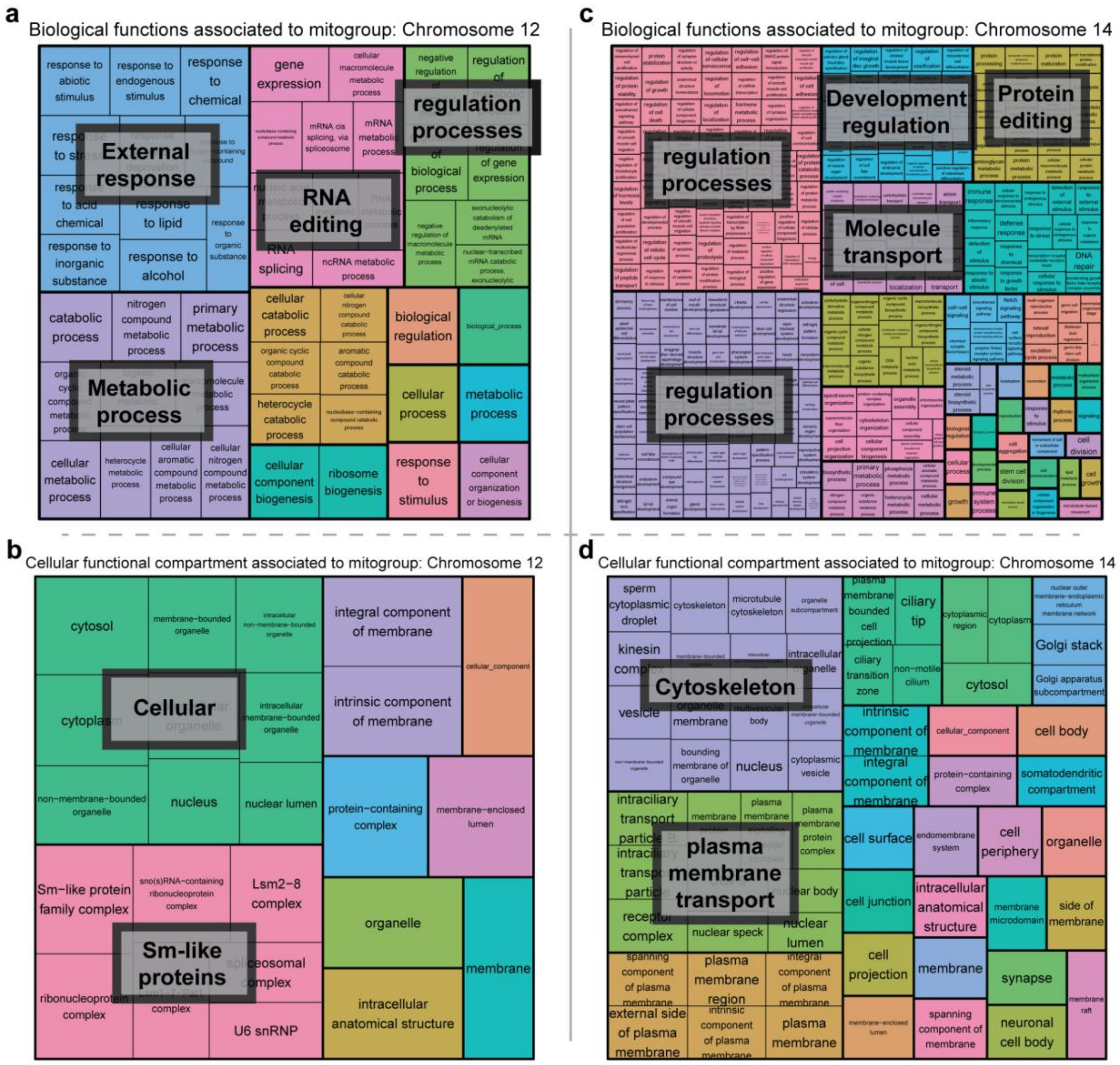

Supplementary Figure 16: Biological functions and Cellular compartments found interacting between mitochondrial and nuclear genomes. a: Biological functions and **b:** Cellular functional compartments of genes found in the region with abundant F_{ST} outliers of chromosome 12. **c:** Biological functions and **d:** Cellular functional compartments of genes found in the region with abundant F_{ST} outliers of chromosome 14. Note that colors do not stand for the same function in different panels.

SUPPLEMENTARY TABLES

Supplementary Table 1: Sample information. For each sample, we provide its Sample code, Locality, Geographic region, coordinates, and mitochondrial COI haplogroup as reported in previous studies. The reference genome was found to be of haplogroup 1.

Sample code	Locality	Geographic region	Latitude (UTM)	Longitude (UTM)	Haplogroup
Ref_DNA_Styela	Barcelona, Spain	Mediterranean	41.38	2.18	1
Ref_RNA_Styela	Barcelona, Spain	Mediterranean	41.38	2.18	1
NC11	North Carolina, USA	Atlantic	34.14	-77.86	2
NC18	North Carolina, USA	Atlantic	34.14	-77.86	2
NC23	North Carolina, USA	Atlantic	34.14	-77.86	2
NC25	North Carolina, USA	Atlantic	34.14	-77.86	2
BRA10	Santa Catarina, Brazil	Atlantic	-26.77	-48.66	2
BRA11	Santa Catarina, Brazil	Atlantic	-26.77	-48.66	2
BRA16	Santa Catarina, Brazil	Atlantic	-26.77	-48.66	1
BRA26	Santa Catarina, Brazil	Atlantic	-26.77	-48.66	1
PE4	Port Elizabeth, South Africa	Atlantic	-33.96	25.64	1
PE7	Port Elizabeth, South Africa	Atlantic	-33.96	25.64	2
PE8	Port Elizabeth, South Africa	Atlantic	-33.96	25.64	1
PE10	Port Elizabeth, South Africa	Atlantic	-33.96	25.64	2
FE6	Ferrol, Spain	Atlantic	43.48	-8.24	1
FE8	Ferrol, Spain	Atlantic	43.48	-8.24	1
FE12	Ferrol, Spain	Atlantic	43.48	-8.24	1
FE22	Ferrol, Spain	Atlantic	43.48	-8.24	2
MIS3	Misaki, Japan	Pacific	35.16	139.62	2
MIS6	Misaki, Japan	Pacific	35.16	139.62	2
MIS17	Misaki, Japan	Pacific	35.16	139.62	2
MIS22	Misaki, Japan	Pacific	35.16	139.62	1
CA3	California, USA	Pacific	32.73	-117.2	2
CA4	California, USA	Pacific	32.73	-117.2	1
CA8	California, USA	Pacific	32.73	-117.2	2
CA9	California, USA	Pacific	32.73	-117.2	1

Supplementary Table 2: Sequencing approaches followed to obtain the reference genome of *Styela plicata*. Library type, tissue used, number of raw reads in millions (M),number of raw bases in Gigabases (Gb), number of filtered reads and number of filtered bases. Raw data can be accessed from BioProjects PRJEB67507 and PRJEB67519.

Sample ID	Library Type	Tissue DNA	RAW reads (M)	RAW bases (Gb)	Filtered reads (M)	Filtered bases (Gb)
Ref_DNA_Styela	Pacbio CLR	Mantle	22.44	180.05	1.19	46.17
	Illumina WGS-SR	Mantle	169.94	30.08	166.61	24.75
	Illumina Omni-C	Mantle	317.21	47.58	308.39	45.72
Ref_RNA_Styela	Illumina RNAseq	Tissue pool	220.10	33.01	107.20	16.08

Supplementary Table 3: Genome assembly statistics. Comparison of the newly generated assembly of *Styela plicata* with four other tunicate species. BUSCO values are provided using a "Complete [Single, Duplicated], Fragmented, Missing" format. Note that the low BUSCO values of *O. dioica* are due to extensive genome reduction, not a low-quality assembly.

**Assembly**	***Styela plicata***	***Styela clava***	***Botryllus schlosseri***	***Ciona intestinalis* A *(C. robusta)***	***Oikopleura dioica***
Accession code	ERZ21873893	GCF_013122585	GCA_051294915	GCA_000224145	GCA_907165135
# scaffolds	1077	211	410	1272	19
# scaffolds (>= 25000 bp)	155	185	315	287	13
# scaffolds (>= 50000 bp)	64	134	176	154	11
Total length (>= 25000 bp)	413425667	339982718	492015640	106688020	64235295
Total length (>= 50000 bp)	410266168	338155010	487040391	101843710	64165187
Total length	419162242	340489724	493670271	115227301	64281565
Estimated genome size	430000000	406780000	725000000	196000000	70000000
GC (%)	35.44	35.28	40.53	35.67	41.05
N50	25036768	20774986	28942301	5152901	16158756
NG50	24821409	20303285	26494593	81022	14533022
N75	22624068	19273745	25542243	396326	12959145
NG75	22624068	14156018	25542243	396326	12959145
L50	8	8	8	9	2
LG50	9	10	12	95	3
L75	13	12	12	27	4
LG75	13	15	13	-	4
# N's per 100 Kbp	86.58	16.39	4.31	2659.67	33.56
BUSCO values	C:92.3%[S:87.7%,D:4.4%],F:3.8%, M:3.9%	C:89.7%[S:83.1%,D:6.6%],F:4.2%, M:6.1%	C:90.3%[S:87.8%,D:2.5%],F:3.8%, M:5.9%	C:93.6%[S:93.0%, D:0.6%],F:2.4%,M:4.0%	C:58.4%[S:55.6%, D:2.8%],F:10.5%, M:31.1%

Supplementary Table 4: Transposable elements (TE) groups: number, length, and percentage in the assembly.

TE Level 1	TE Level 2	TE Level 3	Number of elements	Length of assembly (bp)	Percentage of assembly (%)
Retroelements		Other Retroelements	41055	16610312	3.96
	SINEs	Other SINEs	802	236534	0.06
		Penelope	4737	953233	0.23
	LINEs	Other LINEs	28389	9903690	2.36
		CRE/SLACS	301	87559	0.02
		L2/CR1/Rex	17217	6135601	1.46
		R1/LOA/Jockey	312	205488	0.05
		R2/R4/NeSL	994	685312	0.16
		L1/CIN4	341	260671	0.06
	LTR elements	Other LTR elements	11864	6470088	1.54
		BEL/Pao	4380	2579526	0.62
		Ty1/Copia	567	329200	0.08
		Gypsy/DIRS1	6634	3533917	0.84
DNA transposons	Other DNA-transposons		51358	10162718	2.42
	hobo-Activator		31370	5677841	1.35
	Tc1-IS630-Pogo		1487	459486	0.11
	PiggyBac		245	30371	0.01
	Tourist/Harbinger		5621	1388004	0.33
	Other		5	4240	0.00
Rolling-circles			2763	342607	0.08
Unknown			808048	149638405	35.70
Total interspersed repeats				176411435	42.09
Small RNA			41608	8696664	2.07
Satellites			105	14652	0.00

Simple repeats			44188	2612288	0.62
Low complexity			7655	482671	0.12
**Total repetitive regions**				**188560317**	**44.99**

Supplementary Table 5: Mapping to reference genome. For each sample, we provide the tissue for DNA extraction, the number of raw reads in millions (M), filtered and mapped reads, and mean coverage.

Sample ID	Sequencing platform	Tissue DNA	RAW reads (M)	Filtered reads (M)	Mapped Reads (M)	Mapped Reads	Mean coverage
Ref_DNA_Styela	PacBio	Gill	22.44	1.19	1.18	99.4%	78.5
Ref_DNA_Styela	Illumina WGS	Gill	169.94	166.61	166.5	99.9%	58.2
BRA10	Illumina WGS	Mantle	39.0	39.0	38.5	98.7%	13.0
BRA11	Illumina WGS	Mantle	36.3	36.2	35.8	99.0%	12.1
BRA16	Illumina WGS	Mantle	35.7	35.5	35.2	99.2%	11.9
BRA26	Illumina WGS	Mantle	33.0	32.9	32.6	98.9%	11.0
CA3	Illumina WGS	Mantle	37.6	37.6	37.1	98.6%	12.5
CA4	Illumina WGS	Mantle	31.7	31.5	31.3	99.3%	10.6
CA8	Illumina WGS	Mantle	38.6	38.5	38.2	99.2%	12.9
CA9	Illumina WGS	Mantle	37.5	37.5	37.2	99.1%	12.6
FE6	Illumina WGS	Mantle	37.7	32.5	31.3	96.3%	10.7
FE8	Illumina WGS	Mantle	38.2	34.9	33.3	95.4%	11.3
FE12	Illumina WGS	Mantle	32.6	37.8	37.4	99.2%	12.7
FE22	Illumina WGS	Mantle	34.9	38.1	29.2	76.6%	9.8
MIS3	Illumina WGS	Mantle	38.0	32.6	32.3	99.2%	10.9
MIS6	Illumina WGS	Mantle	34.6	35.2	34.9	99.2%	11.8
MIS17	Illumina WGS	Mantle	32.8	38.1	37.8	99.2%	12.8
MIS22	Illumina WGS	Mantle	35.3	34.5	34.2	99.2%	11.6
NC11	Illumina WGS	Mantle	33.2	33.3	32.8	98.6%	10.9
NC18	Illumina WGS	Mantle	38.4	38.7	38.2	98.6%	12.8
NC23	Illumina WGS	Mantle	36.1	36.2	35.7	98.8%	11.8
NC25	Illumina WGS	Mantle	33.7	33.8	33.4	98.8%	11.1
PE4	Illumina WGS	Mantle	35.2	35.1	34.8	99.3%	11.8
PE7	Illumina WGS	Mantle	38.7	35.0	34.7	99.2%	11.8
PE8	Illumina WGS	Mantle	37.7	38.6	38.3	99.2%	13.0
PE10	Illumina WGS	Mantle	35.2	37.4	37.1	99.1%	12.6

Supplementary Table 6: Linked regions area of influence detected with iDIG, Linkage disequilibrium (LD) and F_{ST}.

	**iDIG area of influence**		**LD area of influence**		**F_{ST} area of influence**	
**Chromosome**	**position 5' (bp)**	**position 3' (bp)**	**position 5' (bp)**	**position 3' (bp)**	**position 5' (bp)**	**position 3' (bp)**
Chromosome 2	2,619,820	15,753,413	2,147,305	14,760,253	2,119,820	16,203,413
Chromosome 4	2,495,369	15,892,631	1,951,322	16,179,388	1,895,369	16,269,236
Chromosome 11	4,415,678	9,485,543	3,589,471	9,468,435	3,555,678	9,885,946
Chromosome 16	6,315,678	14,485,148	5,127,248	13,432,090	5,779,766	14,958,823

Supplementary Table 7: Figure 5b code legend.

**tRNA**		**Coding genes**		**rRNA**	
**Code**	**Gene**	**Code**	**Gene**	**Code**	**Gene**
T	Threonine	1	COX2	s	12S small subunit
D	Aspartic Acid	2	NAD1	l	16S large subunit
G	Glycine	3	NAD4L		
C	Cysteine	4	CYTB		
A	Alanine	5	COX1		
S	Serine	6	NAD6		
M	Methionine	7	NAD2		
L	Leucine	8	NAD5		
R	Arginine	9	NAD3		
N	Asparagine	10	COX3		
W	Tryptophan	11	ATP6		
V	Valine	12	ATP8		
Y	Tyrosine	13	NAD4		
I	Isoleucine				
F	Phenylalanine				
H	Histidine				
K	Lysine				
Q	Glutamine				
E	Glutamic Acid.				
